# Elucidation of Substantial Differences in Ring-Opening Polymerization Outcomes from Subtle Variation of Glucose Carbonate-Based Monomer Substitution Patterns and Substituent Types

**DOI:** 10.1021/jacs.3c03339

**Published:** 2023-07-06

**Authors:** Yidan Shen, Mingwan Leng, Yunchong Yang, Senthil Kumar Boopathi, Guorong Sun, Karen L. Wooley

**Affiliations:** ^†^Departments of Materials Science & Engineering, ^‡^Chemistry, and ^§^Chemical Engineering, Texas A&M University, College Station, Texas 77842, United States

## Abstract

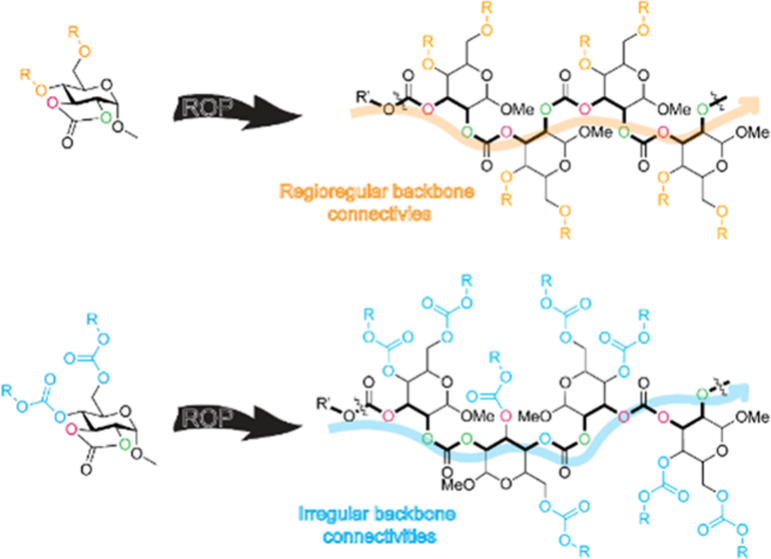

The substituents present upon five-membered bicyclic glucose carbonate monomers were found to greatly affect the reactivities and regioselectivities during ring-opening polymerization (ROP), which contrast in significant and interesting ways from previous studies on similar systems, while also leading to predictable effects on the thermal properties of the resulting polycarbonates. Polymerization behaviors were probed for a series of five five-membered bicyclic 2,3-glucose-carbonate monomers having 4,6-ether, -carbonate, or -sulfonyl urethane protecting groups, under catalysis with three different organobase catalysts. Irrespective of the organobase catalyst employed, regioregular polycarbonates were obtained via ROP of monomers with ether substituents, while the backbone connectivities of polymers derived from monomers with carbonate protecting groups suffered transcarbonylation reactions, resulting in irregular backbone connectivities and broad molar mass distributions. The sulfonyl urethane-protected monomers were unable to undergo organobase-catalyzed ROP, possibly due to the acidity of the proton in urethane functionality. The thermal behaviors of polycarbonates with ether and carbonate pendant groups were investigated in terms of thermal stability and glass transition temperature (*T*_g_). A two-stage thermal decomposition was observed when *tert*-butyloxycarbonyl (BOC) groups were employed as protecting side chains, while all other polycarbonates presented high thermal stabilities with a single-stage thermal degradation. *T*_g_ was greatly affected by side-chain bulkiness, with values ranging from 39 to 139 °C. These fundamental findings of glucose-based polycarbonates may facilitate the development of next-generation sustainable highly functional materials.

## Introduction

Increasing efforts are being devoted toward the development of polymers derived from sustainably sourced feedstocks,^1−6^ which have environmental and technological benefits, while also allowing for an in-depth study of unique polymerization chemistries, leading to highly functional polymer materials. Among these eco-friendly macromolecules, polymers based upon monosaccharides,^7−11^ oligosaccharides,^12,13^ and polysaccharides^14−17^ have gained significant attention due to the great abundance and relatively low cost of natural carbohydrate resources. Moreover, the high degrees of structural diversity and functionality of sugars allow for incorporation of varied side-chain groups/substituents for tailoring the physicochemical properties of the resulting sugar-based polymers.

A key parameter for any polymer is its thermal behavior, and even for recently developed, naturally sourced building blocks, tuning of side-chain substituents has been shown to be a simple approach to modulate the glass transition temperature (*T*_g_). For instance, Coates, DiStasio, and co-workers investigated the relationship between thermal properties and substituents of polyesters derived from sugar-based furan derivatives, in which polymers with moderate steric hindrance exhibited the highest *T*_g_ values.^18^ In another example, Wu, Wang, and co-workers found that the side-chain structural rigidities greatly affected the *T*_g_s of bio-based polyesters obtained from ring-opening copolymerizations of tetracyclic anhydrides with different epoxides.^19^ We also demonstrated that the *T*_g_ values of poly(4,6-α-d-glucose carbonate)s (4,6-PGC), prepared through ring-opening polymerizations (ROPs) of six-membered bicyclic 4,6-glucose carbonate monomers, could be tuned by varying the moieties at the 2- and 3-positions of the corresponding glucose carbonate monomers.^20^

In addition to effects on the thermal characteristics, it was noted that side-chain substituents also influenced the regiochemistry during the 1,5,7-triazabicyclo[4.4.0]dec-5-ene (TBD)-catalyzed ROP of these bicyclic 4,6-glucose carbonates.^20−22^ Indeed, it is well known that the nature of substituent functionality plays crucial roles in affecting kinetics and other polymerization behaviors.^23−26^ Comprehensive studies for the six-membered bicyclic 4,6-glucose carbonates were conducted,^27^ which revealed highly regioregular backbone connectivities in the case of monomers containing carbonate protecting groups at the 2- and 3-positions, whereas those protected as ethers resulted in formation of regioirregular polycarbonate backbones. This result was intriguing, given that the carbonate side-chain functionalities seemed to invoke the regioregularity during ROP without becoming directly involved in transcarbonylation reactions.

**Figure 1:**
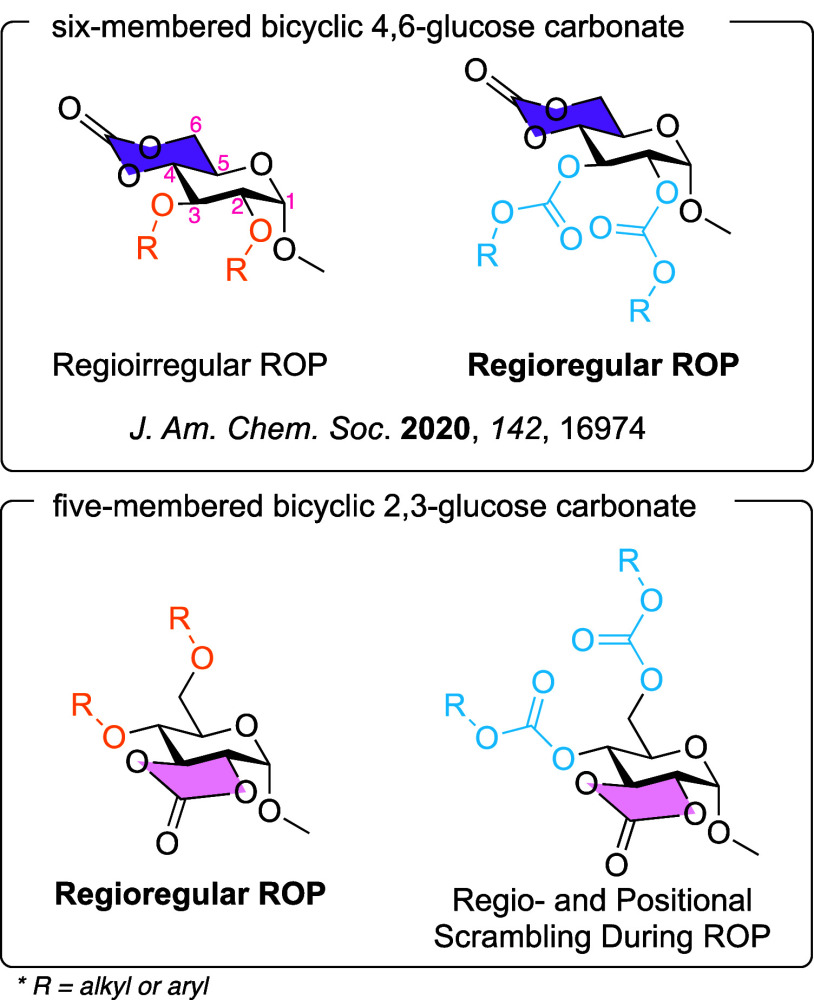
Past and current 4,6- versus 2,3-glucose bicyclic carbonate monomer structures having ether versus carbonate substituents in the 2,3- versus 4,6-positions, respectively, with designation of their unusual regiochemical outcomes following ROPs. *The purple highlight was the six-membered carbonate ring, and the pink highlight was the five membered carbonate ring.

This current study, therefore, aimed to investigate these interesting phenomena, expand the scope, and gain an understanding of the fundamental principles. Five-membered bicyclic 2,3-glucose carbonate monomers with either ether or carbonate linkages on the 4- and 6-positions served as positional isomers to the six-membered bicyclic 4,6-glucose carbonate monomers (Figure 1).^27^ Interestingly, the monomer substituent effects on ROPs leading to poly(2,3-α-d-glucose carbonate)s (2,3-PGC) revealed an opposite trend in the backbone connectivity to the corresponding 4,6-PGCs. In contrast to the analogous 4,6-PGCs, ether substituents led to significant degrees of regioregularity. This result was also in contrast to TBD-catalyzed ROPs of five-membered tricyclic 2,3-glucose carbonate monomers containing cyclic acetal groups through the 4- and 6-positions,^28^ which experienced complexities of regioselective ring-opening versus transcarbonylation-driven structural metamorphosis. As may be expected, in this current work, the bicyclic 2,3-glucose carbonate monomers having carbonate substituents suffered significant transcarbonylation/scrambling. Moreover, the side-chain substituents were also shown to affect the thermal properties of the final polycarbonate materials produced.

## Results and Discussion

### Monomer Design and Syntheses

**Scheme 1:**
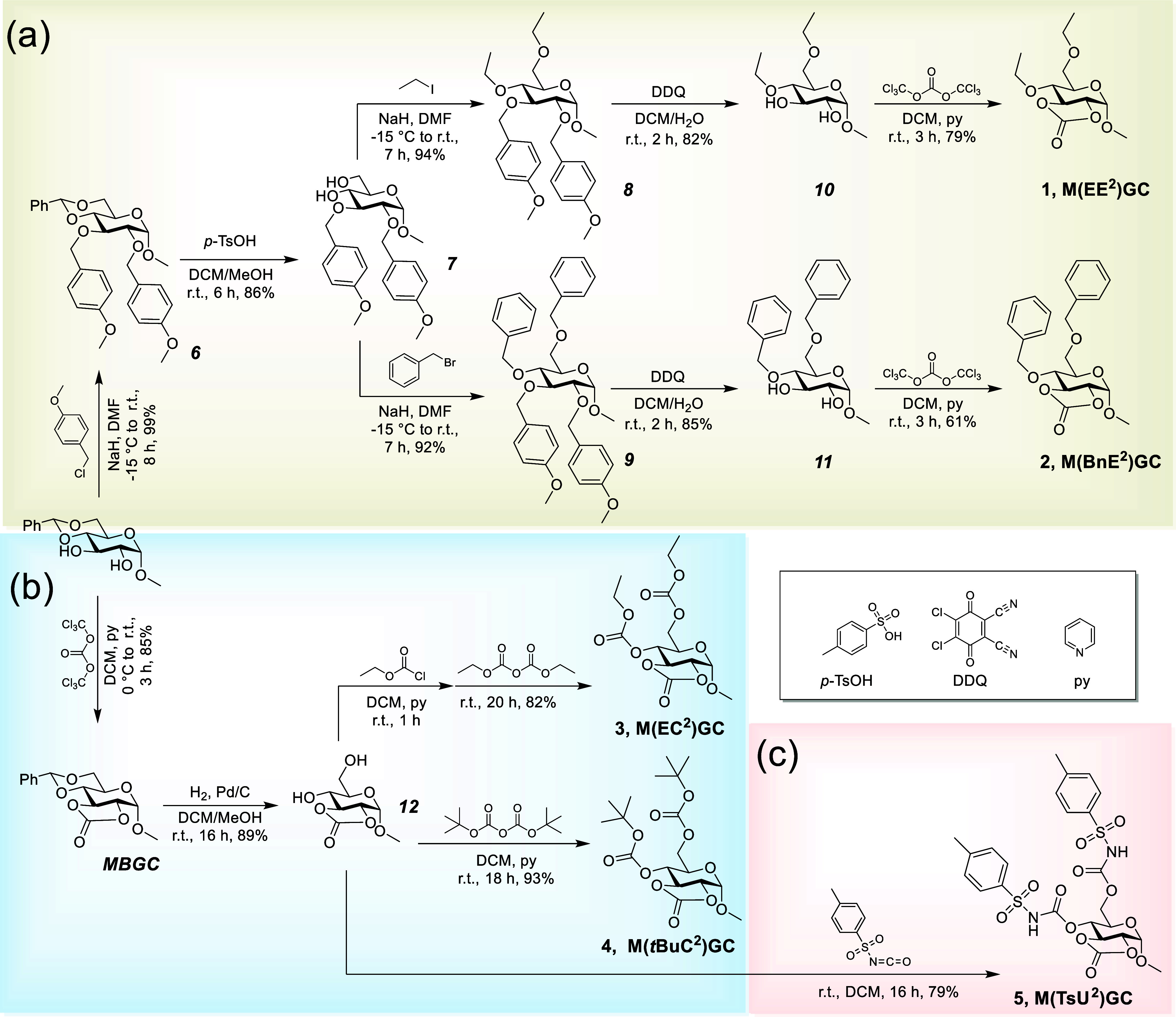
Synthesis of Bicyclic Glucose Carbonate Monomers (a) with Ether Substituents **1** and **2**, (b) with Carbonate Substituents **3** and **4**, and (c) with Sulfonyl Urethane Substituents **5**

As shown in Scheme 1, three different side-chain functionalities, i.e., ether, carbonate, and sulfonyl urethane, were introduced to the 4- and 6-positions of 1-methyl-α-d-glucopyranoside. Although a broad range of ether and carbonate substituents had been investigated for the previous six-membered bicyclic 4,6-glucose carbonate monomer systems,^20,27^ here, we selected ethyl and benzyl ethers and ethyl and *tert*-butyl carbonates as representative examples that would be able to probe the steric and electronic effects of the side-chain substituents during ROPs and their influence on the properties of the resulting polymers.

The installation of ether side chains (Scheme 1a) involved five-step reaction sequences that began with “temporary” protection of the 2,3-hydroxyl positions and concluded by the cyclic carbonate being formed in the final step.^29^ Protection of commercially available 1-methyl-4,6-benzylidene-α-d-glucopyranoside by the reaction with *p*-methoxybenzyl chloride was followed by the selective removal of the benzylidene protecting group under acidic conditions, which gave **7** with free hydroxyl groups at the 4- and 6-positions. Ethylation or benzylation of **7** under basic conditions provided compounds **8** and **9**, respectively. The *p*-methoxybenzyl groups were oxidatively removed by using 2,3-dichloro-5,6-dicyano-1,4-benzoquinone (DDQ), affording monomer precursors **10** and **11**, respectively. Finally, phosgenation of the 2,3-hydroxyl groups in **10** and **11** resulted in 1-methyl-4,6-di-*O*-ethyl/benzyl-2,3-*O*-carbonyl-α-d-glucopyranoside monomers [**M(EE**^**2**^**)GC**, **1** and **M(BnE**^**2**^**)GC**, **2**, respectively].

The syntheses of monomers with carbonate substituents (Scheme 1b) were relatively straightforward. Cyclic carbonylation of 1-methyl-4,6-benzylidene-α-d-glucopyranoside furnished 1-methyl-4,6-benzylidene-2,3-*O*-carbonyl-α-d-glucopyranoside (**MBGC**).^30^ After hydrogenolysis of **MBGC**, the 4,6-carbonate side-chain groups were introduced onto **12** using dialkyl dicarbonate with/without pretreatment using the corresponding alkyl chloroformate to obtain **M(EC**^**2**^**)GC** (**3**) and **M(*****t*****BuC**^**2**^**)GC** (**4**), respectively. Interestingly, for the production of **3**, both diethyl dicarbonate and ethyl chloroformate were required. In the absence of diethyl dicarbonate, the major products were monosubstituted, predominantly at the 6-position; however, upon reaction with only diethyl dicarbonate, no ethyl carbonate-substituted products were observed. Optimized conditions were identified to be sequential addition of ethyl chloroformate followed by diethyl dicarbonate in a one-pot reaction in dichloromethane (DCM) in the presence of pyridine at room temperature to afford **3** in 82% yield. In contrast, synthesis of **4** was accomplished in 93% yield using only di-*tert*-butyl dicarbonate and pyridine in DCM at room temperature.

### Organocatalytic ROPs, Kinetic Study, and Polymer Structures

**Scheme 2:**
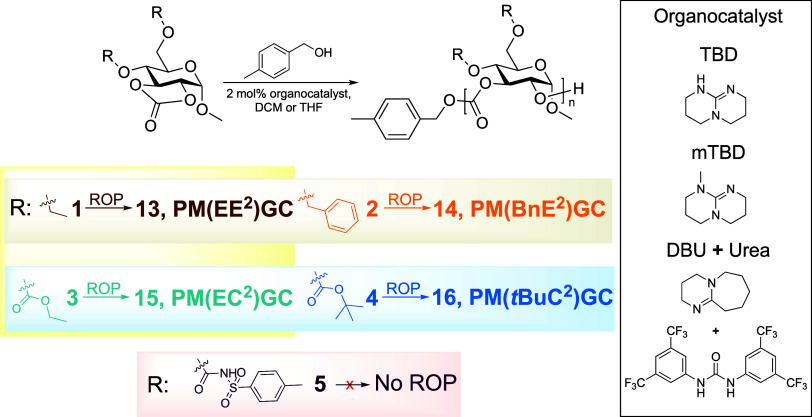
Synthesis of Polycarbonates via Organobase-Catalyzed ROPs

The organocatalytic ROPs of a series of five-membered bicyclic 2,3-glucose carbonate monomers (**1**–**4**) were investigated using TBD,^31−33^ 7-methyl-1,5,7-trizazabicyclo-[4.4.0]dec-5-ene (mTBD),^34,35^ or a binary system comprising 1,8-diazabicyclo(5.4.0)undec-7-ene (DBU) and 3,5-bis(trifluoromethyl)phenyl urea (Urea),^36−38^ as organobase catalysts (Scheme 2 and Table 1). Among the surveyed organobase catalysts, the reactivity order was as follows: TBD > mTBD > DBU + Urea.

**Table 1:** Details for ROP Experiments at Room Temperature Using Five-Membered Bicyclic 2,3-Glucose Carbonate Monomers, **1**–**5**, with Ether, Carbonate, or Sulfonyl Urethane Groups at the 4- and 6-Positions via Organobase Catalysis with Initiation by 4-Methylbenzyl Alcohol

entry	catalyst^a^	monomer^a^	time^c^	*M*_n_ (kDa)^e^	*Đ*^e^	yield^f^
1	TBD	**1**, M(EE^2^)GC	7 h	13.4	1.08	89%
2	TBD	**2**, M(BnE^2^)GC	27 h	13.9	1.19	95%
3	TBD	**3**, M(EC^2^)GC	27 h	3.82	1.90	48%
4	TBD	**4**, M(tBuE^2^)GC	49 h	2.69	2.76	33%
5	TBD	**5**, M(TsU^2^)GC	20 h^d^	0.38	n/a	0%
6	mTBD	**1**, M(EE^2^)GC	10 d	13.2	1.13	90%
7	mTBD	**2**, M(BnE^2^)GC	14 d	15.6	1.14	97%
8	mTBD	**3**, M(EC^2^)GC	30 h	12.1	1.13	89%
9	mTBD	**4**, M(*t*BuE^2^)GC	7 d	12.0	1.21	93%
10	mTBD	**5**, M(TsU^2^)GC	20 h^d^	0.38	n/a	0%
11	DBU + Urea^b^	**1**, M(EE^2^)GC	17 d	5.94	1.16	60%
12	DBU + Urea^b^	**2**, M(BnE^2^)GC	20 d	5.38	1.19	69%
13	DBU + Urea^b^	**3**, M(EC^2^)GC	11 d	4.62	1.22	55%
14	DBU + Urea^b^	**4**, M(*t*BuE^2^)GC	15 d	4.03	1.23	52%
15	DBU + Urea^b^	**5**, M(TsU^2^)GC	20 h^d^	0.38	n/a	0%

a Each ROP was conducted at a monomer-to-initiator-to-catalyst molar ratio of 50:1:1.

b Molar ratio of DBU to Urea was 1:1.

c Reaction times were recorded once conversion exceeded 95%.

d For **5**, no conversion was observed at 20 h.

e *M*_n_ and *Đ* were measured by SEC using THF as the eluent and calibrated using linear polystyrene standards.

f Yields were measured gravimetrically from isolated polymer samples, with each polymerization having been formed to conversions >95%.

**Figure 2:**
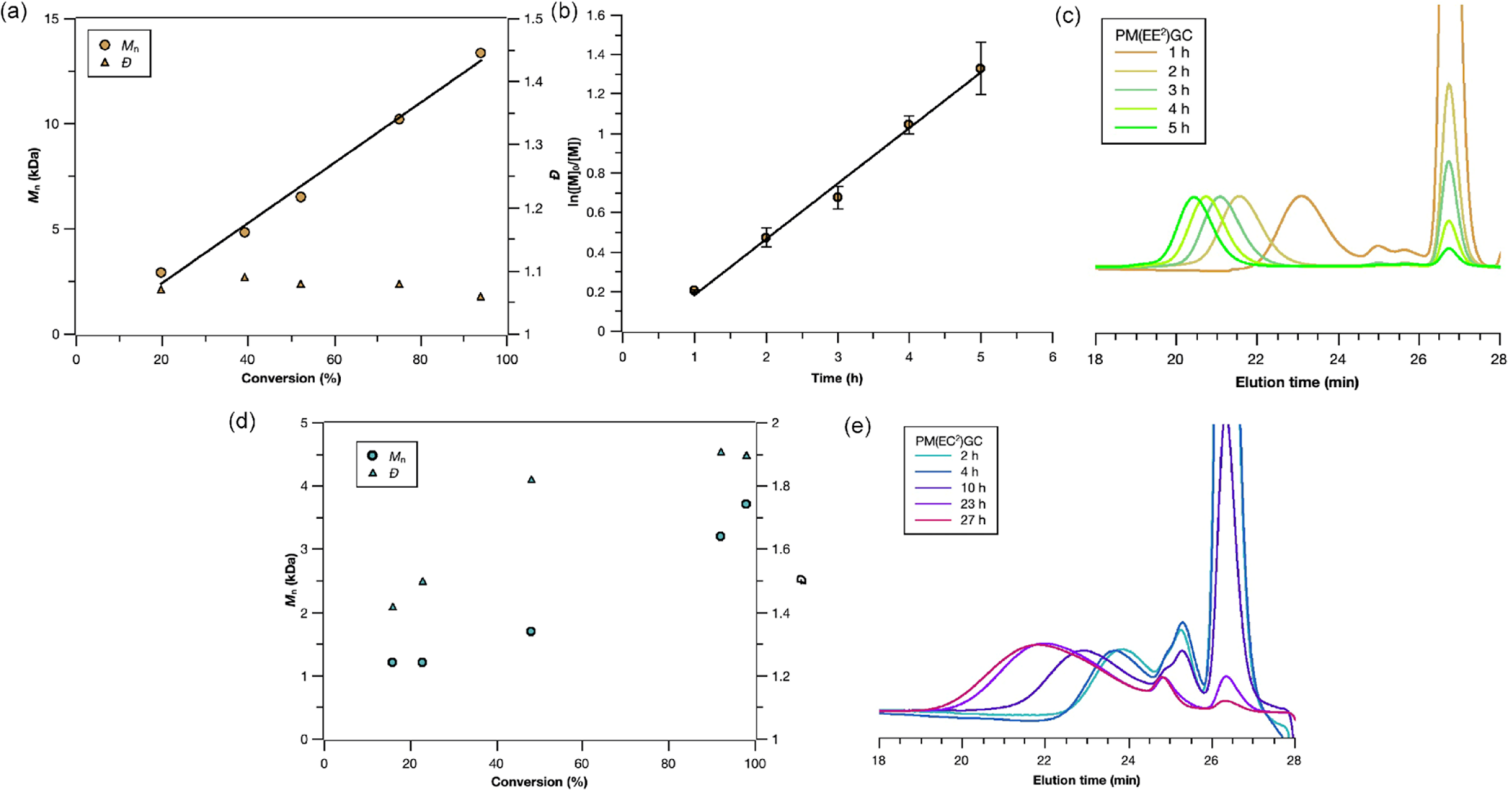
Plot of *M*_n_ and *Đ* as a function of monomer conversion (%) for the polymerization of (a) **1** or (d) **3** using TBD as the catalyst. The ratio of [monomer ]_0_/[ initiator ]_0_/[TBD]_0_ was 50:1:1. (b) Kinetic plots of monomer conversion (ln([*M*]_0_/[*M*])) as a function of time using data obtained by SEC (RI detector). SEC traces (THF as the eluent, 1 mL/min) of the ROP of (c) **1** or (e) **3** as a function of polymerization time, with normalization of the intensity of the polymer peaks.

Although polycarbonates were afforded from all four of the ether- or carbonate-4,6-functionalized monomers, **1**–**4**, there were significant differences in the extent of control in the ROPs. The ROPs of **1**–**4** initiated by 4-methylbenzyl alcohol (MBA) at room temperature in DCM with a fixed monomer/initiator feed ratio of 50:1 were initially studied under TBD catalysis (Table 1, entries 1–4), with monitoring of monomer conversion and polymer chain growth as a function of time by size exclusion chromatography (SEC Table 1). For consistency, determinations of conversion for all polymerization were made by SEC, rather than by NMR, as the carbonate side-chain polymers had overlapping and broad ^1^H NMR signals, leading to inaccuracies (Figure S40). The SEC traces showed unimodal peaks during the polymerization of **1** or **2** containing ether side chains (Figures 2c and S7b), which shifted toward earlier elution times as the reactions proceeded. The molar masses grew linearly with monomer conversions (Figures 2a and S7a) while maintaining consistently narrow dispersity values (*Đ* < 1.2). The kinetic plot of ln([M]_0_/[M]) versus time, processed for monomer **1** bearing ethyl ether substituents, was linear (Figure 2b), revealing first-order kinetics and control during the ROP process. In contrast, the products from ROPs of **3** or **4** exhibited broad and multimodal SEC traces throughout the progression of the reactions (Figures 2e and S8b). Moreover, the experimental *M*_n_ values did not grow linearly with monomer conversions, and the *Đ* values increased significantly as the polymerizations progressed (Figures 2d and S8a). As a note, all *M*_n_ and *Đ* values were calculated by including the elution time range from the onset of the growing polymer peak to 25.75 min, to capture the full spectrum of polymeric to oligomeric species. These differences indicate that the relatively controlled growth for the ether side-chain polymers was contrasted by the loss of control in the carbonate side-chain analogues. The lack of control and broad dispersities were later revealed by NMR studies (vide infra) to be due to transcarbonylation reactions involving the side-chain carbonates. Though different ring-opening characteristics were observed, the polymerization rates showed similar trends in each subclass of substituted monomers, in which monomers with bulkier substituents exhibited qualitatively slower ROP rates.

**Figure 3:**
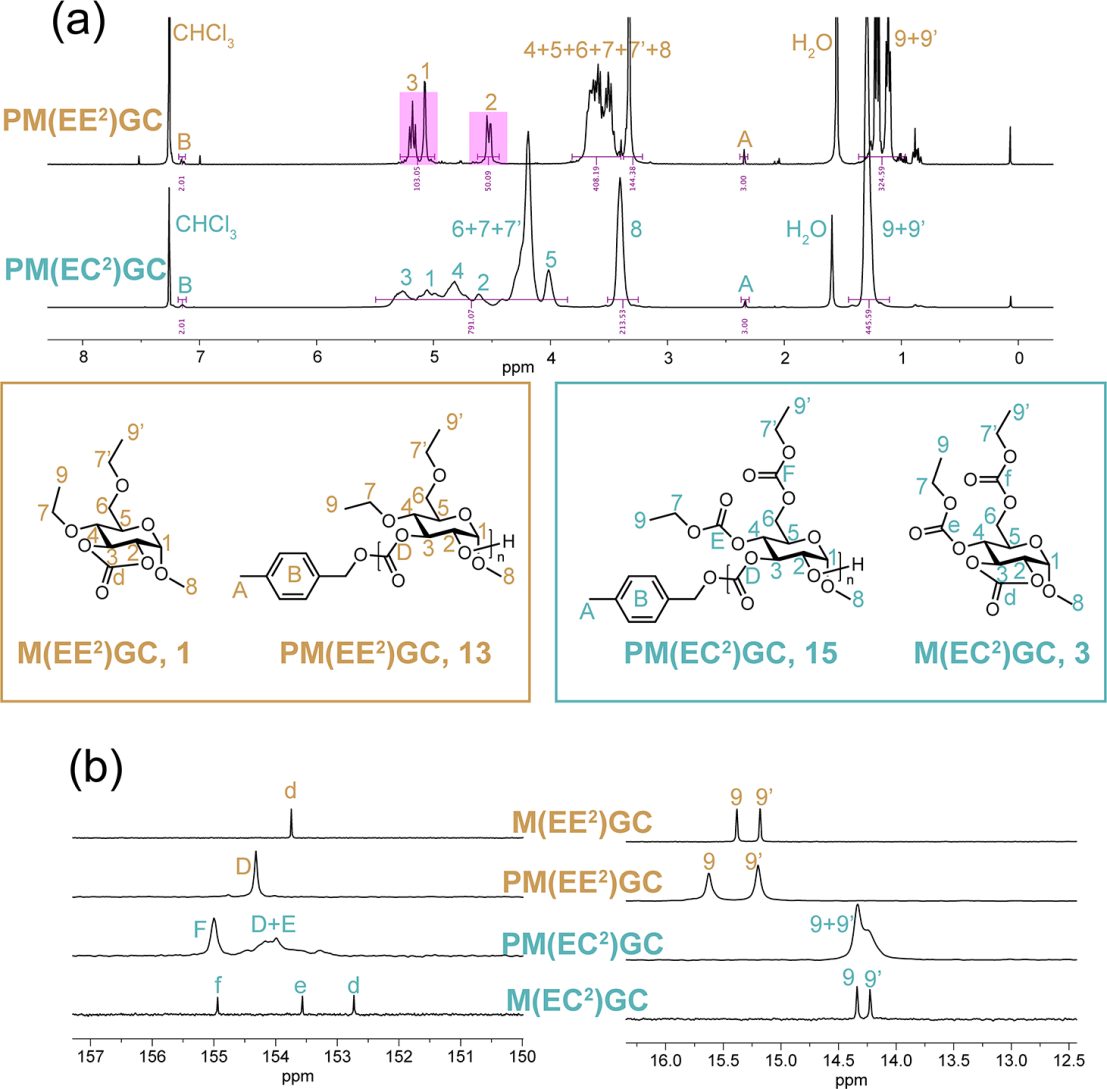
(a) ^1^H NMR spectra (500 MHz) of PM(EE^2^)GC, **13** (top), and PM(EC^2^)GC, **15** (bottom), and (b) the partial ^13^C NMR spectra (126 MHz) of **1**, **13**, **15**, and **3** (from top to bottom) in CDCl_3_ presenting carbonyl carbon, C_9_ and C_9′_ resonances. *The full spectra can be found in Figure S38. **Polymers were prepared via TBD-catalyzed ROP.

The polymer structures were rigorously investigated by FT-IR, ^1^H NMR, and ^13^C NMR spectroscopies. Both PM(EE^2^)GC (**13**) and PM(BnE^2^)GC (**14**) protected by ethers showed characteristic C=O stretching bands at 1759 cm^–1^, at a slightly lower frequency than that for monomer cyclic carbonate carbonyls (1807 cm^–1^), corresponding to the carbonate backbone functionalities. The carbonyl signals (C_D_) for these polymers resonated at *ca.* 154.2 ppm in the ^13^C NMR spectra (Figures 3b and S17b), which were shifted downfield relative to the carbonyl signals (C_d_) of their corresponding monomers. For each **13** and **14**, the presence of a single ^13^C carbonate signal indicated uniformity in the backbone structure, i.e., high degrees of regioregularities. Achieving a regioregular polymer backbone requires a preferred acyl-oxygen bond cleavage during ROP. This finding is in contrast to the acetal-protected five-membered tricyclic 2,3-glucose carbonates^28^ and the ether-protected six-membered bicyclic 4,6-glucose carbonate monomers^27^ from previous studies, each of which showed regioirregularity. However, this regioregularity is in agreement with the observed regioselective ring opening for a six-membered tricyclic 4,6-mannose carbonate having isopropylidene protection at the 2- and 3-positions reported by Buchard et al.^38^ and for carbonate-protected six-membered bicyclic 4,6-glucose carbonate monomers with alkyloxycarbonyl at the 2- and 3-positions from our earlier work.^27^ The well-defined proton signals and clear splitting patterns in the ^1^H NMR spectra of **13** and **14** (pink highlight, Figures 3a and S17a), in conformity with ^13^C NMR analyses, also suggested a regiochemical preference, either 2-to-3 (head-to-tail) or 3-to-2 (tail-to-head).

To reveal the ring-opening preference during the polymerizations, unimer fractions were isolated from the equimolar experiment between **1** or **2** and MBA in the presence of 2 mol % TBD (Figure S19), and their structures were probed using ^1^H NMR spectroscopy (Figure S20). Two unimer isomers were observed, resonating as two sets of proton signals within each spectrum. In each case, the ratio of the two unimers was the same (21:79). Due to their different polarities, separation using column chromatography allowed for structure identification by 1D NMR combined with homonuclear and heteronuclear 2D NMR measurements (Figures S21–S28). For **1** and **2**, the preferred ring-opening site occurred by cleavage at the C–O2 bond, yielding the methylbenzyl carbonate at the 3-position and the hydroxyl group at the 2-position of the sugar ring for the unimer having ethyl or benzyl ether protecting groups. Therefore, the preferred ring-opening direction would be 2-to-3, involving C–O2 bond cleavage during the ROPs and affording polycarbonates having a majority head-to-tail regioregularity.

In contrast, the regiochemical outcomes were ill-defined for ROPs of the monomers having carbonate protecting groups, **3** and **4**. Broad and merged peaks in ^1^H and ^13^C NMR spectra of PM(EC^2^)GC, **15** (Figure 3a,b, respectively) and PM(*t*BuC^2^)GC, **16** (Figure S18a,b, respectively) provided initial insights into the regioirregular polymer structures. Detailed examination revealed the involvement of the side-chain carbonates, further complicating the backbone regiochemistry beyond the simple directionality preference during ring opening. Most noteworthy were the analyses of the ^13^C carbonate signals of the cyclic carbonates (C_d_) and the side-chain carbonates at the 4- (C_e_) and 6-positions (C_f_) of the monomers versus the carbonate ^13^C NMR resonances identified as being due to the carbonyls of carbonate functionalities along the backbone (C_D_) and at the 4-position (C_E_) and 6-position (C_F_) of the polymers. The C_F_ signals of **15** and **16** were maintained as relatively sharp peaks after polymerization, resonating as single distinct signals from the other carbonyls and at frequencies that were similar to the C_f_ of their respective monomers, **3** and **4**. These results indicated that the 6-position carbonate groups were less likely to be involved in side reactions during ROP. The C_D_ and C_E_ resonances of **15** and **16**, however, presented as multiple broad overlapping signals, indicating regioirregular ring opening and/or transcarbonylation during the ROPs, which seemed to involve combinations of the 2-, 3- and 4-positions. ROP-related transcarbonylation side reactions were noticed in our previous study between the 2- and 3-positions of five-membered tricyclic 2,3-carbonate monomers^28^ and also reported in a range of cyclic carbonate monomers bearing carbonate and carbamate side chains.^39^

The carbonate substituent installations at the 4- and 6- positions increased the possible transferable sites for the carbonate groups. Proposed intermolecular or intramolecular transcarbonylation mechanisms are shown in Figures S36 and S37. With the potentials for involvement of the 2-, 3-, 4-, and 6-positions in inter/intramolecular transcarbonylation reactions, there would be 12 possible isomers from the unimer fraction derived from **3** or **4** (Figure S29). Unfortunately, the unimer fractions failed to be chromatographically separable, thereby preventing confirmation of this hypothesis.

To reduce the potential involvement of the side-chain functionalities in transcarbonylation reactions during ROP, sulfonyl urethane groups were investigated (Scheme 1c). Accordingly, sulfonyl urethane-substituted monomer **M(TsU**^**2**^**)GC** (**5**) was obtained directly by the condensation reaction of *p*-toluenesulfonyl isocyanate with hydroxyl groups of common intermediate **12**. As a note, *p*-toluenesulfonyl isocyanate was utilized due to the fact that reactions between the 4-hydroxyl group of **12** and “conventional” alkyl isocyanates were inefficient (typically, the desired di-substituted product was afforded in less than 10% yield after several days, data not shown). This approach, however, failed when attempted ROPs of monomer **5** did not result in the production of polymers even after 20 h, as monitored by SEC (Figure S6). This phenomenon may be attributed to the acidity of the sulfonyl urethane proton, which can scavenge the organobase catalyst.^40−42^ For instance, Rubin and co-workers reported that the sulfonyl urethane ethyl-*p*-toluenesulfonyl carbamate has a p*K*_a_ of 3.7, comparable to that of acetic acid.^43^ As a consequence, the organobase catalysts (0.02 equiv) were likely to undergo acid–base reactions and be quenched by the acidic sulfonyl urethane side chains (2 equiv).

Consequently, less active organic bases, mTBD, and a cocatalyst system of DBU+Urea were also employed as catalysts for ROP. Due to the poor solubility of 3,5-bis(trifluoromethyl)phenyl urea in DCM, the ROPs involving cocatalysts were conducted in THF. Nevertheless, during ROP of **M(TsU**^**2**^**)GC**, **5** using less active organocatalysts, no polymer products were observed as monitored by SEC (Figure S6). In contrast, the employment of 2 mol % mTBD or the cocatalyst system successfully triggered the polymerization of monomers **1**–**4** to obtain the corresponding polycarbonates (Table 1, entries 6–9 and 11–14, respectively). The molar masses of polymers **13**–**16** prepared in the presence of the DBU + Urea cocatalyst were lower than the expected molar masses corresponding to the conversion, which was suspected to be the result of DBU having served as a zwitterionic initiator in the ROP and was supported by MALDI-TOF mass spectrometry showing a cyclic species (Figure S34c, orange star).^44,45^ Meanwhile, the urea catalyst was not fully removed from the polymer chain after precipitation purification (Figure S34c, pink star).

A qualitative comparison of the kinetics of ROP for each monomer using different catalysts demonstrated that less active catalysts resulted in slower polymerization rates with better control. ROPs of ether-substituted monomers **1** and **2** were well controlled with their dispersity values being less than 1.2 (Figures S9, S10, S13, and S14), irrespective of the organocatalyst employed. The nearly identical ^1^H and ^13^C NMR spectra of polymers **13** and **14** prepared using different catalysts (Figures S30 and S31) indicated that the regiochemical preference remained the same during ROP. Interestingly, ROPs of carbonate-substituted monomers **3** and **4** with less active organocatalysts presented better-controlled polymerizations, as indicated by their relatively narrow dispersities (Figures S11, S12, S15, and S16). Particularly, during the DBU+Urea catalytic polymerization, the linear molar mass (*M*_n_) growth of the polymer with monomer conversion and unimodal distributions were observed by SEC. Further, NMR analyses of polymers **15** and **16** prepared under conditions of DBU+Urea catalysis (Figures S32 and S33) showed sharper carbonyl C_E_ resonances having less complicated component features, implying fewer occurrences of transcarbonylation involving the 4-position than those observed using more reactive TBD or mTBD catalysts.

### Thermal Properties of Glucose-Based Polycarbonates

**Figure 4:**
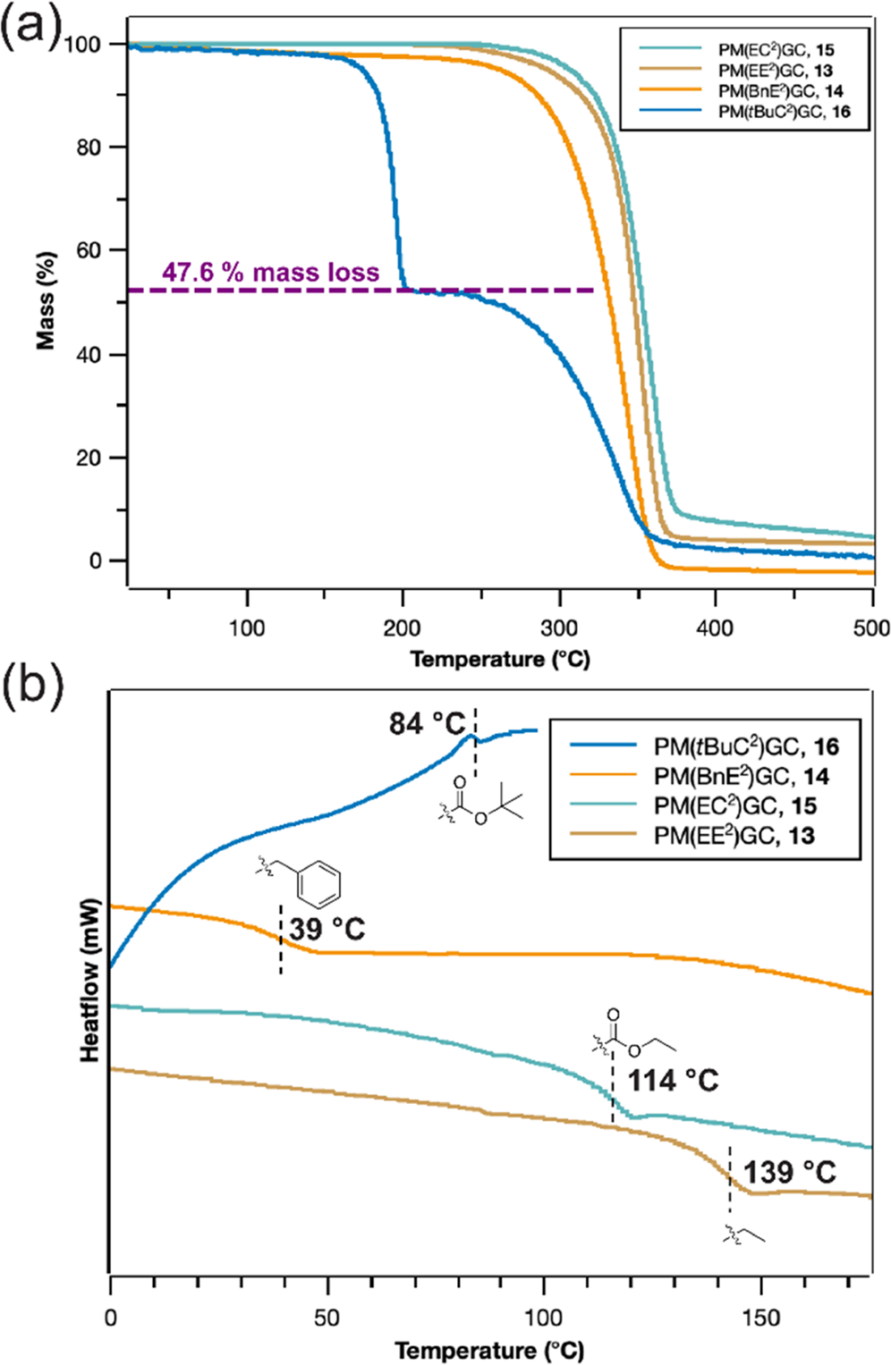
(a) TGA thermograms and (b) DSC thermograms of polymers **13**–**16**.

Polycarbonates with ether or carbonate side chains that had nearly equal molar masses were prepared for investigation and comparison of thermal behaviors (Figure S35), ensuring that thermal property differences would arise primarily from their side-chain compositions. The thermal stabilities of polycarbonates with various side-chain functionalities as measured by thermogravimetric analysis (TGA) are presented in Figure 4a. A two-stage thermal degradation was observed uniquely, only for polymer **16**. Quantitative analysis indicated that the first thermal degradation stage, proceeding from 160 to 200 °C, involved BOC group removal, with a mass loss of *ca.* 48%, agreeing well with the theoretical thermal decomposition of BOC groups per repeating unit (47.6%, Figure 4a, as indicated by the reference purple dashed line). The second stage of thermal decomposition then proceeded for the backbone over the range of *ca.* 250–350 °C, with a thermal decomposition profile similar to those single-stage mass losses observed for the other three polycarbonates, **13**–**15**. The differential scanning calorimetry (DSC) traces are presented in Figures 4b and S39, showing the range of *T*_g_ values obtained for a series of polycarbonates bearing various side-chain functionalities. The DSC analyses of polymers **13**–**15** were conducted with thermal cycles run from −50 to 150 °C, whereas polymer **16** was probed only up to 100 °C to avoid the BOC group thermal degradation. On increasing the side-chain bulkiness from ethyl ether to benzyl ether, *T*_g_ underwent a remarkable 100 °C reduction, from 139 to 39 °C. Similarly, the polymer having ethyl carbonate versus bulkier BOC side chains gave a 30 °C difference, 114 versus 84 °C, respectively.

## Conclusions

ROPs of a new family of five-membered bicyclic 2,3-glucose-carbonate monomers were investigated using different organobase catalysts embarking on studies to expand the understanding of carbohydrate-derived cyclic carbonate monomers, polymers, and their properties. It was expected that these investigations would simply fill in gaps of knowledge. However, surprises were encountered. In contrast to earlier work, 2,3-glucose carbonate monomers having ether substituents at the 4- and 6-positions offered regioregular polycarbonates from ROPs conducted at room temperature. It is worthwhile to note that an ultralow temperature and carbonate protecting groups were required to achieve a high degree of polymer regioregularity and control during the ROPs of the six-membered 4,6-glucose carbonates, as reported earlier.^27^ Although the observation of transcarbonylations upon the carbonate side-chain protecting groups during polymerizations that scrambled the ring-opening processes of this report may be expected, the regioregularity of the ether-substituted analogues was not anticipated. Temperature may have played a role during the ROPs, but it could not be the only factor. Beyond the ROP differences observed as a function of side-chain chemistry, the thermal behaviors of 2,3-PGCs having varied acyclic side chains were found to differ across a wide range of glass transition temperatures, from 39 to 139 °C.

As investigated herein, subtle substituent differences between monomers significantly affected the ROP behaviors and the polymer properties. The transcarbonylation side reactions alongside the ROPs, which extended from the ring-opening sites to also involve distal groups in the carbohydrate framework, may be considered to be a negative outcome. However, combinations and control over such chemical transformations may be utilized to diversify polymer structures through in situ structural metamorphosis. These methodology developments and physicochemical property findings provide an in-depth understanding of composition–structure–topology–morphology–property relationships for the family of 2,3-PGCs, paving the way for the future employment of glucose and other carbohydrates as renewable feedstocks for sustainable engineering materials.

## Supporting Information Available

Experimental procedures; computational details; ^1^H NMR; ^13^C NMR; COSY; HSQC; HMBC; SEC; and MALDI-mass (PDF)
