# Supplementary material for: Elucidation of Substantial Differences in Ring-Opening Polymerization Outcomes from Subtle Variation of Glucose Carbonate-Based Monomer Substitution Patterns and Substituent Types
Source: J Am Chem Soc. 2023 Jul 6;145(28):15405–13. doi: 10.1021/jacs.3c03339 (PMC10863030; doi:10.1021/jacs.3c03339)
Supplement: Supplementary file 1 — ja3c03339_si_001.pdf [file ja3c03339_si_001.pdf]

Supporting Information for

Advances in substituent effects on the ring-opening polymerizations and thermal behaviors of glucose-based polycarbonates

Yidan Shen,† Mingwan Leng,‡ Yunchong Yang,† Senthil Kumar Boopathi,‡ Guorong Sun,‡ and Karen L. Wooley*,†,‡,§

†Departments of Materials Science & Engineering, ‡Chemistry, and §Chemical Engineering, Texas A&M University, College Station, Texas 77842, USA

*Corresponding author e-mail: wooley@chem.tamu.edu

Table of Contents

I. Experimental Procedures and Characterization Data 5

Instrumentation, Methods and Analysis 5

II. Figures 14

Figure S1. ***^{1}H NMR (500 MHz) and ^{13}C NMR (126 MHz) spectra of M(EE[2])GC (1) in $CDCl_3$.*** 14

Figure S2. ***^{1}H NMR (500 MHz) and ^{13}C NMR (126 MHz) spectra of M(BnE[2])GC (2) in $CDCl_3$.*** 15

Figure S3. ***^{1}H NMR (500 MHz) and ^{13}C NMR (126 MHz) spectra of M(EC[2])GC (3) in $CDCl_3$.*** 16

Figure S4. ***^{1}H NMR (500 MHz) and ^{13}C NMR (126 MHz) spectra of M(tBuC[2])GC (4) in $CDCl_3$.*** 17

Figure S5. ***^{1}H NMR (500 MHz) and ^{13}C NMR (126 MHz) spectra of M(TsU[2])GC (5) in $CDCl_3$.*** 18

Figure S6. ***ROPs of M(TsU[2])GC, 5 with different catalysts after 20 h.*** 19

Figure S7. ***(a) Plot of M_n and Đ as a function of monomer conversion (%) for the polymerization of M(BnE[2])GC, 2 using TBD as catalyst. (b) SEC traces (THF as eluent, 1mL/min) of the ROP as a function of polymerization time, with normalization of the intensity of the polymer peaks.*** 19

Figure S8. ***(a) Plot of M_n and Đ as a function of monomer conversion (%) for the polymerization of M(tBuC[2])GC, 4 using TBD as catalyst. (b) SEC traces (THF as eluent, 1mL/min) of the ROP as a function of polymerization time, with normalization of the intensity of the polymer peaks.*** 20

Figure S9. ***(a) Plot of M_n and Đ as a function of monomer conversion (%) for the polymerization of M(EE[2])GC, 1 using mTBD as catalyst. (b) SEC traces (THF as eluent, 1mL/min) of the ROP as a function of polymerization time, with normalization of the intensity of the polymer peaks.*** 20

Figure S10. ***(a) Plot of M_n and Đ as a function of monomer conversion (%) for the polymerization of M(BnE[2])GC, 2 using mTBD as catalyst. (b) SEC traces (THF as eluent, 1mL/min) of the ROP as a function of polymerization time, with normalization of the intensity of the polymer peaks.*** 21

Figure S11. ***(a) Plot of M_n and Đ as a function of monomer conversion (%) for the polymerization of M(EC[2])GC, 3 using mTBD as catalyst. (b) SEC traces (THF as eluent, 1mL/min) of the ROP as a function of polymerization time, with normalization of the intensity of the polymer peaks.*** 21

Figure S12. ***(a) Plot of M_n and Đ as a function of monomer conversion (%) for the polymerization of M(tBuC[2])GC, 4 using mTBD as catalyst. (b) SEC traces (THF as eluent, 1mL/min) of the ROP as a function of polymerization time, with normalization of the intensity of the polymer peaks.*** 22

Figure S13. (a) Plot of M_n and Đ as a function of monomer conversion (%) for the polymerization of M(EE^2)GC, 1 using DBU+Urea as catalyst. (b) SEC traces (THF as eluent, 1mL/min) of the ROP as a function of polymerization time, with normalization of the intensity of the polymer peaks. 22

Figure S14. (a) Plot of M_n and Đ as a function of monomer conversion (%) for the polymerization of M(BnE^2)GC, 2 using DBU+Urea as catalyst. (b) SEC traces (THF as eluent, 1mL/min) of the ROP as a function of polymerization time, with normalization of the intensity of the polymer peaks. 23

Figure S15. (a) Plot of M_n and Đ as a function of monomer conversion (%) for the polymerization of M(EC^2)GC, 3 using DBU+Urea as catalyst. (b) SEC traces (THF as eluent, 1mL/min) of the ROP as a function of polymerization time, with normalization of the intensity of the polymer peaks. 23

Figure S16. (a) Plot of M_n and Đ as a function of monomer conversion (%) for the polymerization of M(tBuC^2)GC, 4 using DBU+urea as catalyst. (b) SEC traces (THF as eluent, 1mL/min) of the ROP as a function of polymerization time, with normalization of the intensity of the polymer peaks. 24

Figure S17. ^{1}H NMR (500 MHz) and ^{13}C NMR (126 MHz) spectra of PM(BnE^2)GC (14) via TBD-catalytic ROP in $CDCl_3$ 24

Figure S18. ^{1}H NMR (500 MHz) and ^{13}C NMR (126 MHz) spectra of PM(tBuC^2)GC (16) via TBD-catalytic ROP in $CDCl_3$ 25

Figure S19. (a) Scheme of unimer preparation, MAlDI-TOF spectrum of unimers of (b) M(EE^2)GC, 7 and (c) M(BnE^2)GC, 8. 25

Figure S20. ^{1}H NMR spectrum of a crude mixture of the unimeric isomeric isomers (a) of M(EE^2)GC and (b) of M(BnE^2)GC. 26

Figure S21. ^{1}H NMR, COSY and HSQC spectrum of M(EE^2)GC unimer regioisomer II in $CDCl_3$. 27

Figure S22. HMBC spectrum of M(EE^2)GC unimer regioisomer II in $CDCl_3$. 28

Figure S23. ^{1}H NMR, COSY and HSQC spectrum of M(EE^2)GC unimer regioisomer I in $CDCl_3$. 29

Figure S24. HMBC spectrum of M(EE^2)GC unimer regioisomer I in $CDCl_3$. 30

Figure S25. ^{1}H NMR, COSY and HSQC spectrum of M(BnE^2)GC unimer regioisomer II in $CDCl_3$. 31

Figure S26. HMBC spectrum of M(BnE^2)GC unimer regioisomer II in $CDCl_3$. 32

Figure S27. ^{1}H NMR, COSY and HSQC spectrum of M(BnE^2)GC unimer regioisomer I in $CDCl_3$. 33

Figure S28. HMBC spectrum of M(EE^2)GC unimer regioisomer I in $CDCl_3$. 34

Figure S29. Possible unimer structures of M(EC^2)GC with the occurrences of intra- and intermolecular transcarbonylation reactions. 35

Figure S30. Comparison of ^{1}H NMR (500 MHz) and ^{13}C NMR (126 MHz) spectra of PM(EE^2)GC (13) via ROP using different catalyst in $CDCl_3$ 36

Figure S31. Comparison of ^{1}H NMR (500 MHz) and ^{13}C NMR (126 MHz) spectra of PM(BnE^{2})GC (14) via ROP using different catalyst in $CDCl_3$. 36

Figure S32. Comparison of ^{1}H NMR (500 MHz) and ^{13}C NMR (126 MHz) spectra of PM(EC^{2})GC (15) via ROP using different catalyst in $CDCl_3$. 37

Figure S33. Comparison of ^{1}H NMR (500 MHz) and ^{13}C NMR (126 MHz) spectra of PM(tBuC^{2})GC (16) via ROP using different catalyst in $CDCl_3$. 37

Figure S34. MALDI-TOF MS spectra of PM(EE^{2})GC via organocatalytic ROP using (a) TBD, (b) mTBD, or (c) DBU+Urea. 38

Figure S35. SEC traces of polymers for thermal analyses. 39

*Figure S36. The initiation step of the ROP of 2,3-a-d-glucose carbonate, catalyzed by (a) TBD, (b) mTBD, and (c) DBU+Urea and initiated by MBA. R = side chain protecting group. (*There are supposed to be four possible pathways of the initiation.[1, 2] Only the initiator and catalyst attacked syn to the anomeric methoxy group that involved C-O2 cleavage was presented.)* 40

Figure S37. (a) 2- and 3- position intramolecular transcarbonylation, (b) 2- and 3- position intermolecular transcarbonylation, (c) side chain involved intramolecular transcarbonylation, (d) side chain involved intermolecular transcarbonylation. 41

Figure S38. ^{13}C NMR (126 MHz) spectra of 1, 13, 15, and 3 (from top to bottom) in $CDCl_3$. The resonances in dashed box were enlarged in Figure 3(b). 42

Figure S39. An enlarged DSC of Polymer 16. 42

Figure S40. (a) ^{1}H NMR of PM(EE^{2})GC and M(EE^{2})GC, (b) ^{1}H NMR of PM(EC^{2})GC and M(EC^{2})GC. 43

References 43

I. Experimental Procedures and Characterization Data

Instrumentation, Methods and Analysis

^{1}H NMR, ^{13}C NMR, (^{1}H-^{1}H) COSY, (^{13}C-^{1}H) HSQC, and (^{13}C-^{1}H) HMBC spectra were recorded on Bruker 400 with an Ascend magnet, an automated tuning 5 mm broadband iProbe, and a 60 position SampleXpress sample changer, or Avance 500 with an Oxford magnet, an automated tuning 5 mm ^{1}H/^{13}C/^{15}N cold probe, and a 24 position SampleCase sample changer interfaced to a UNIX computer using the VnmrJ software. Chemical shifts for ^{1}H NMR and ^{13}C NMR signals were referenced to the solvent resonance frequencies.

Fourier transform-infrared (FT-IR) spectra were recorded on an IR Prestige 21 system, equipped with a diamond attenuated total reflection (ATR) lens (Shimadzu Corp., Japan), and analyzed using IRsolution *v*. 1.40 software.

Size exclusion chromatography (SEC) eluting with tetrahydrofuran (THF) was conducted on a Waters chromatography, Inc. (Milford, MA) system equipped with an isocratic pump model 1515, a differential refractometer model 2414, and a Four-column set including a guard column (PLgel 5 µm, 50 × 7.5 mm) and three Styragel columns (PLgel 5 µm Mixed C, 500 Å, and 10^4 Å, 300 × 7.5 mm columns). The system was operated at 40 °C with a flow rate of 1 mL/min. Data were analyzed using Breeze software from Waters Chromatography, Inc. (Milford, MA). Molar masses were determined relative to polystyrene standards (300–467,000 Da) purchased from Polymer Laboratories, Inc. (Amherst, MA). Polymer solutions were prepared at a concentration of *ca*. 3-8 mg/mL with 0.05 vol% toluene as a flow rate marker and an injection volume of 200 µL was used.

Preparative size exclusion chromatography (prep SEC) eluting with chloroform was conducted on a JAI LC-9230II NEXT Chromatography, Inc. (Japan) system equipped with a reciprocating double plunger pump (model P-9104B), a UV-vis 4ch NEXT detector at four wavelengths (254 nm, 280 nm, 300 nm, 330 nm), and a two-column set, including a JAIGEL-H 40P Guard column and a JAIGEL-2H-40 HPLC column. The system was equilibrated at room temperature with chloroform at the flow rate 14.0 mL/min. Data collection and analysis were performed with JAI Scan™ software. Polymer solutions were prepared at a concentration of *ca.* 10 mg/mL in chloroform and an injection volume of 5 mL was used.

Glass transition temperatures (T_g) were measured by differential scanning calorimetry (DSC) on a Mettler-Toledo DSC3/700/1190 (Mettler-Toledo, Inc., Columbus, OH) under a nitrogen gas atmosphere. Measurements were performed on sample masses of *ca.* 5 – 10 mg in aluminum pans with heating and cooling rates of 10 °C/min at a range of -50 °C – 180 °C (unless otherwise noted), and three heating and cooling cycles were conducted. Measurements were analyzed using Mettler-Toledo STAR^{e} *v*.

15.00a software. The T_g was taken as the midpoint of the inflection tangent of the third heating scan.

Thermogravimetric analysis (TGA) was performed on the sample mass of *ca.* 5 – 10 mg in an aluminum pan under N_2 atmosphere using a Mettler-Toledo TGA2/1100/464, at a range of 25 – 500 °C with a heating rate of 10 °C/min. Data were analyzed using Mettler-Toledo STAR^e *v*. 15.00a software.

Electrospray ionization mass spectrometry (ESI-MS) experiments were performed using a Thermo Scientific Q Exactive Focus. Samples were injected (10 µL) using methanol as a mobile phase at a flow rate of 300 µL/min. The Q Exactive Focus HESI source was operated in full MS in positive mode. The mass resolution was tuned to 70000 FWHM at m/z 200. The spray voltage was set to 3.75 kV, and the sheath gas and auxiliary gas flow rates were set to 7 and 0 arbitrary units, respectively. The transfer capillary temperature was held at 250 °C and the S-Lens RF level was set at 50 v. Exactive Series 2.11/Xcalibur 4.2.47 software was used for data acquisition and processing.

Matrix-assisted laser desorption ionization-time of flight mass spectrometry (MALDI-TOF MS) was performed on a microflex™ LRF mass spectrometer (Bruker Corporation, Billerica, MA) in positive linear mode. Ions were generated by a pulsed nitrogen laser (337 nm, 25 kV), and 200 laser pulses were used per spectrum. Trans-2-[3-(4-tert-butylphenyl)-2-methyl-2-propylidene]malononitrile (DCTB) and potassium trifluoroacetate (KTFA) were used as a matrix and cationization reagent, respectively. The sample and matrix were prepared at 1 and 26 mg/mL, respectively, in chloroform, and KTFA was prepared at 1 mg/mL in acetone. The sample solution was mixed with the matrix and KTFA at a volumetric ratio of 2:2:1, and 1 µL of the mixture was deposited onto a stainless-steel sample holder and dried in air prior to the measurement.

Materials

Methyl 4,6-*O*-benzylidene-α-D-glucopyranoside was used as received from Carbosynth Ltd (Aurora, IL). Triphosgene was used as received from Oakwood Products Inc. (Estill, SC). 1,5,7-Triazabicyclo [4.4.0]dec-5-ene (TBD) was used as received from ChemScene LLC (Monmouth Junction, NJ). 7-Methyl-1,5,7-triazabicyclo [4.4.0]dec-5-ene (mTBD) was used as received from Frontier Scientific (Logan, UT). 3,5-Bis(trifluoromethyl)phenyl isocyanate, 3,5-bis(trifluoromethyl)aniline and 4-methoxybenzyl chloride were used as received from TCI Chemicals Inc. (Portland, OR). Diethyl dicarbonate and 2,3-dichloro-5,6-dicyano-1,4-benzoquinone (DDQ) were used as received from Chem-Impex Inc. (Wood Dale, IL). 1,8-Diazabicyclo(5.4.0)undec-7-ene were purified with distillation as received from Chem-Impex Inc. (Wood Dale, IL). Dichloromethane (DCM), dimethylformamide (DMF), and tetrahydrofuran (THF) were purified by a solvent purification system (J. C. Meyer Solvent Systems, Inc., Laguna Beach, CA). All other chemicals were purchased from Sigma-Aldrich, Co. (St. Louis, MO)

or VWR (Radnor, PA), and were used as received unless otherwise noted. 4-Methylbenzyl alcohol (MBA) was purified by recrystallization from hexanes and ethyl acetate and stored in the glovebox under Ar atmosphere. All monomers were dried under reduced pressure with phosphorous pentoxide (P_2O_5) and stored in the glovebox under Ar atmosphere before polymerization.

Synthetic protocols

Synthesis of methyl 4,6-di-*O*-alkyl/aryl-2,3-*O*-carbonyl-α-D-glucopyranosides (M(RE^2)GC):

Methyl 4,6-*O*-benzylidene-2,3-di-*O*-*p*-methoxybenzyl-α-D-glucopyranoside, *6*:
Methyl 4,6-*O*-benzylidene-α-D-glucopyranoside (21.0 g, 74.4 mmol) was dissolved in anhydrous DMF (200 mL) and stirred at −15 °C, and NaH (11.9 g, 298 mmol, 60% w/w in mineral oil) was added sequentially in five portions every 5 min under N_2. After being stirred for 15 min, *p*-methoxybenzyl chloride (35.1 g, 224 mmol) was added dropwise in *ca*. 20 min. After stirring for another 30 min at −15 °C, the reaction mixture was warmed to room temperature over a period of *ca*. 30 min and stirred for 6 h. The reaction mixture was quenched with cold water and extracted with DCM (4 × 200 mL). The combined organic layer was washed with brine, dried over Na_2SO_4, filtered, and concentrated under reduced pressure. The residue was purified through recrystallization in hexanes (Hex)/ethyl acetate (EA) to give the product as a white solid, ***6*** (38.5 g, 73.7 mmol, yield: 99 %). R_f = 0.4 (Hex:EA = 3:1). ^{1}H NMR (400 MHz, $CDCl_3$) δ ppm 7.49 (m, 2H), 7.41 – 7.34 (m, 3H), 7.29 (dd, *J* = 8.5, 7.0 Hz, 4H), 6.85 (dd, *J* = 8.5, 7.0 Hz, 4H), 5.54 (s, 1H), 4.86 – 4.74 (m, 3H), 4.62 (d, *J* = 12.0 Hz, 1H), 4.53 (d, *J* = 4.0 Hz, 1H), 4.25 (dd, *J* = 10.0, 4.6 Hz, 1H), 4.00 (dd, *J* = 9.4 Hz, *J* = 9.4 Hz, 1H), 3.80 (m, 7H), 3.69 (dd, *J* = 10.0 Hz, 4.6 Hz 1H), 3.57 (dd, *J* = 9.4 Hz, *J* = 12.0 Hz 1H), 3.51 (dd, *J* = 9.4, 4.0 Hz, 1H), 3.39 (s, 3H). ^{13}C NMR (100 MHz, $CDCl_3$) δ ppm 159.55, 159.33, 137.60, 131.07, 130.43, 129.90, 129.85, 129.04, 128.36, 126.18, 113.98, 113.87, 101.39, 99.50, 82.26, 78.86, 78.43, 75.19, 73.56, 69.22, 62.47, 55.49, 55.42, 55.41. FT-IR(ATR) 3188-2674, 1702, 1672, 1605, 1499, 1446, 1348, 1223, 1175, 1062, 1017, 919, 806, 738, 678, 641 cm^{-1}. HRMS (ESI$^+$) Calcd. for (M+H$^+$) $C_{30}H_{34}O_8H^+$ 523.2326; found 523.2317.

Methyl 2,3-di-*O*-*p*-methoxybenzyl-α-D-glucopyranoside, *7*:
To a solution of ***6*** (20.2 g, 38.6 mmol) in DCM/methanol (150 mL, v/v = 1:1), *p*-TsOH (4.96 g, 26.3 mmol) was added. The reaction mixture was stirred at room temperature for 6 h and neutralized with Et_3N (5 mL). The reaction was concentrated under reduced pressure, and the residue was purified by column chromatography (DCM:EA=1:1) to give the product as a viscous transparent colorless liquid, ***7*** (14.4 g, 33.1 mmol, yield: 86%). ^{1}H NMR (400 MHz, $CDCl_3$) δ ppm 7.33-7.27 (m, 4H), 6.92 – 6.85 (m, 4H), 4.94 (d, *J* = 11 Hz, 1H), 4.72 (d, *J* = 11 Hz, 1H), 4.62 (d, *J* = 3.0 Hz, 1H), 4.59 (d, *J* = 3.0 Hz, 1H), 4.54 (d, *J* = 3.5 Hz, 1H), 3.82 – 3.69 (m, 9H), 3.60 (ddd, *J* = 4.0 Hz, *J* = 5.0 Hz, *J* = 10.0 Hz, 1H),

3.49 – 3.43 (m, 2H), 3.37 (s, 3H), 2.16 (d, *J* = 2.5 Hz, 1H), 1.86 (dd, *J* = 7.0, 5.5 Hz, 1H). ^{13}C NMR (100 MHz, $CDCl_3$) δ ppm 159.67, 159.60, 131.03, 130.28, 129.87, 129.77, 114.26, 114.08, 98.47, 81.05, 79.63, 75.11, 72.96, 70.82, 70.73, 62.81, 55.44, 55.41. FT-IR(ATR) 3631-3122, 3070-2745, 1605, 1500, 1447, 1350, 1289, 1228, 1026, 894, 806, 73, 692 cm^{-1}. HRMS (ESI^+) Calcd. for ($M+NH_4^+$) $C_{23}H_{30}O_8NH_4^+$ 452.2279; found 452.2270.

Methyl 4,6-di-*O*-ethyl-2,3-di-*O-p*-methoxybenzyl-α-D-glucopyranoside, *8*:

NaH (1.84 g, 46.0 mmol, 60% w/w in mineral oil) was added sequentially in two portions (every 5 min) to the solution of ***7*** (3.98 g, 9.16 mmol) in DMF (100 mL) at -15 °C under N_2. After stirring at the same temperature for 15 min, CH_3CH_2I (2.2 mL, 28 mmol) in 10 mL DMF was added dropwise. After stirring for another 30 min at −15 °C, the reaction mixture was warmed to room temperature over a period of *ca*. 30 min and stirred for another 6 h, followed by quenching with cold water. The product was extracted with 200 mL DCM twice. The combined organic layer was dried with brine and Na_2SO_4, filtered, and concentrated under reduced pressure. The residue was purified by column chromatography (Hex:EA = 2:1) to give a viscous transparent colorless liquid, ***8***. (4.20 g, 8.57 mmol, yield: 94%). ^{1}H NMR (400 MHz, $CDCl_3$) δ ppm 7.31 – 7.24 (m, 4H), 6.92 – 6.78 (m, 4H), 4.84 (d, *J* = 10.5 Hz, 1H), 4.76 – 4.69 (m, 2H), 4.57 (d, *J* = 12.0 Hz, 1H), 4.52 (d, *J* = 3.5 Hz, 1H), 3.87 – 3.78 (m, 8H), 3.64 – 3.53 (m, 5H), 3.49 – 3.42 (m, 2H), 3.40 - 3.33 (m, 4H), 1.19 (q, *J* = 7.0 Hz, 6H). ^{13}C NMR (101 MHz, $CDCl_3$) δ ppm 159.51, 159.29, 131.38, 130.58, 129.84, 129.71, 113.96, 113.90, 98.47, 81.90, 79.49, 77.82, 75.47, 73.16, 70.22, 69.04, 68.36, 66.95, 55.41, 55.20, 15.92, 15.19. FT-IR(ATR) 3122-2745, 1728, 1596, 1500, 1350, 1279, 1228, 1122, 1079, 1035, 938, 807, 736, 692 cm^{-1}. HRMS (ESI^+) Calcd. for ($M+NH_4^+$) $C_{27}H_{38}O_8NH_4^+$ 508.2905; found 508.2907.

Methyl 4,6-di-*O*-benzyl-2,3-di-*O-p*-methoxybenzyl-α-D-glucopyranoside, *9*:

NaH (0.98 g, 46.0 mmol, 60% w/w in mineral oil) was added sequentially in two portions (every 5 min) to the solution of ***7*** (2.14 g, 4.92 mmol) in DMF (50 mL) at -15 °C under N_2. After stirring at the same temperature for 15 min, (1.8 mL, 17 mmol) in 6 mL DMF was added dropwise. After stirring for another 30 min at −15 °C, the reaction mixture was warmed to room temperature over a period of *ca*. 30 min After stirring for another 30 min at −15 °C, the reaction mixture was warmed to room temperature over a period of *ca*. 30 min and allowed to be stirred for another 6 h, followed by quenching with cold water. The product was extracted with 200 mL DCM twice. The combined organic layer was dried with brine and Na_2SO_4, filtered, and concentrated under reduced pressure. The residue was purified by column chromatography (Hex:EA = 2:1) to give a viscous transparent colorless liquid, ***9***. (2.80 g, 4.56 mmol, yield: 92%). ^{1}H NMR (500 MHz, $CDCl_3$) δ ppm 7.35 – 7.22 (m, 12H), 7.19 – 7.12 (m, 2H), 6.85 (dd, *J* = 8.5, 7.0 Hz, 4H), 4.89 (d, *J* = 10.5 Hz, 1H), 4.84 (d, *J* = 10.5 Hz, 1H), 4.74 (dd, *J* = 11.0, 3 Hz, 2H), 4.63 – 4.55 (m, 3H), 4.48 (d, *J* = 12.0 Hz, 2H), 3.95 (t, *J* = 9.5 Hz, 1H), 3.80 (d, *J* = 4.5 Hz, 6H), 3.76 – 3.68 (m, 2H),

3.66 – 3.57 (m, 2H), 3.52 (dd, *J* = 9.5, 3.5 Hz, 1H), 3.37 (s, 3H). ^{13}C NMR (126 MHz, $CDCl_3$) δ ppm 159.58, 159.34, 138.55, 138.16, 131.26, 130.53, 129.88, 129.78, 128.49, 128.00, 127.93, 127.79, 127.76, 114.03, 113.96, 98.46, 82.04, 79.74, 77.88, 75.56, 75.12, 73.62, 73.18, 70.23, 68.76, 55.43, 55.30. FT-IR(ATR) 3140-2745, 1737, 1605, 1500, 1438, 1394, 1350, 1298, 1228, 1131, 1026, 929, 807, 728, 692 cm^{-1}. HRMS (ESI^+) Calcd. for ($M+Na^+$) $C_{37}H_{42}O_8Na^+$ 637.2772; found 637.2759.

Methyl 4,6-di-*O*-ethyl-α-D-glucopyranoside, *10*:

To a solution of ***8*** (4.13 g, 8.36 mmol) in DCM/H2O (110 mL, v/v = 10:1), DDQ (11.24 g, 49.51 mmol) was added at room temperature. The reaction was stirred for 2 h and then quenched by 100 mL $NaHCO_3$ (aq). The product was extracted by 200 mL DCM five times. The combined organic layer was dried over Na_2SO_4, filtered, and concentrated under reduced pressure. The residue was purified by column chromatography (DCM/EA = 1:1) to give a white solid ***10*** (1.71 g, 6.84 mmol, yield: 82%). ^1H NMR (500 MHz, $CDCl_3$) δ ppm 4.79 (d, *J* = 4.0 Hz, 1H), 3.87 – 3.79 (m, 1H), 3.75 (td, *J* = 9.5, 2.5 Hz, 1H), 3.71 – 3.58 (m, 5H), 3.57 – 3.47 (m, 2H), 3.41 (s, 3H), 3.34 (t, *J* = 9.5 Hz, 1H), 2.50 (d, *J* = 2.5 Hz, 1H), 2.05 (d, *J* = 9.5 Hz, 1H), 1.22 (dt, *J* = 10.0, 7.0 Hz, 6H). ^{13}C NMR (126 MHz, $CDCl_3$) δ ppm 99.27, 77.55, 75.33, 72.75, 70.45, 69.04, 68.23, 67.06, 55.42, 15.89, 15.23. FT-IR(ATR) 3587-3112, 3090-2719, 1736, 1447, 1412, 1359, 1201, 1122, 1035, 929, 895, 833, 745, 692 cm^{-1}. HRMS (ESI^+) Calcd. for ($M+Na^+$) $C_{11}H_{22}O_6Na^+$ 273.1309; found 273.1309.

Methyl 4,6-di-*O*-benzyl-α-D-glucopyranoside, *11*:

To a solution of ***9*** (2.75 g, 4.47 mmol) in DCM/H2O (110 mL, v/v = 10:1), DDQ (6.17 g, 27.2 mmol) was added at room temperature. The reaction was stirred for 2 h and then quenched by 60 mL $NaHCO_3$ (aq). The product was extracted by 200 mL DCM three times. The combined organic layer was dried over Na_2SO_4, filtered, and concentrated under reduced pressure. The residue was purified by column chromatography (DCM/EA = 1:1) to give a white solid ***11*** (1.42 g, 3.79 mmol, yield: 85%). ^1H NMR (500 MHz, $CDCl_3$) δ ppm 4.79 (d, *J* = 4.0 Hz, 1H), 3.87 – 3.79 (m, 1H), 3.75 (td, *J* = 9.5, 2.5 Hz, 1H), 3.71 – 3.58 (m, 5H), 3.57 – 3.47 (m, 2H), 3.41 (s, 3H), 3.34 (dd, *J* = 9.5 Hz, *J* = 9.4 Hz 1H), 2.50 (d, *J* = 2.5 Hz, 1H), 2.05 (d, *J* = 9.5 Hz, 1H), 1.22 (dt, *J* = 10.0, 7.0 Hz, 6H). ^{13}C NMR (126 MHz, $CDCl_3$) δ ppm 99.27, 77.55, 75.33, 72.75, 70.45, 69.04, 68.23, 67.06, 55.42, 15.89, 15.23. FT-IR(ATR) 3105-2763, 1719, 1508, 1394, 1350, 1254, 1201, 1122, 1042, 929, 701cm^{-1}. HRMS (ESI^+) Calcd. for ($M+H^+$) $C_{21}H_{26}O_6H^+$ 375.1802; found 375.1797.

Methyl 4,6-di-*O*-ethyl-2,3-*O*-carbonyl-α-D-glucopyranoside (M(EE^2)GC), 1:

In a flame-dried 250 mL round bottom flask charged with a stir bar, ***10*** (1.32 g, 5.27 mmol) was dissolved in anhydrous DCM with pyridine (2.0 mL, 25 mmol), followed by the addition of triphosgene (0.81 g, 2.7 mmol) in 5 mL DCM. The reaction was allowed to stir at room temperature for 3 h and quenched by $NaHCO_3$(aq). The organic layer was extracted and washed with HCl (5%) solution and brine in sequence and then dried over

Na_2SO_4. DCM was removed under reduced pressure, and the residue was purified by column chromatography (gradient Hex/EA) to afford a viscous light yellow liquid **1** (1.15 g, 4.17 mmol, yield: 79%). ^{1}H NMR (400 MHz, $CDCl_3$) δ ppm 5.14 (d, *J* = 3.0 Hz, 1H), 4.72 (dd, *J* = 11.5, 10 Hz, 1H), 4.17 (dd, *J* = 11.5, 3.0 Hz, 1H), 3.90 – 3.77 (m, 2H), 3.73 – 3.46 (m, 9H), 1.21 (q, *J* = 7.0 Hz, 6H). ^{13}C NMR (101 MHz, $CDCl_3$) δ ppm 153.75, 95.81, 80.35, 77.37, 75.27, 72.64, 68.02, 67.25, 66.92, 55.98, 15.39, 15.19. FT-IR(ATR) 3052-2736, 1807, 1438, 1368, 1254, 1069, 1000, 939, 877, 771, 719, 692 cm^{-1}. HRMS (ESI$^+$) Calcd. for ($M+Na^+$) $C_{12}H_{20}O_7Na^+$ 299.1101; found 299.1102.

Methyl 4,6-di-*O*-benzyl-2,3-*O*-carbonyl-α-D-glucopyranoside (M(BnE^2)GC), 2:

In a flame-dried 250 mL round bottom flask charged with a stir bar, ***11*** (1.32 g, 3.53 mmol) was dissolved in anhydrous DCM with pyridine (1.4 mL, 17 mmol), followed by the addition of triphosgene (0.52 g, 1.7 mmol) in 5 mL DCM. The reaction was allowed to stir at room temperature for 3 h and quenched by $NaHCO_3$(aq). The organic layer was extracted and washed with HCl (5%) solution and brine in sequence and then dried over Na_2SO_4. DCM was removed under reduced pressure, and the residue was purified by column chromatography (gradient Hex/EA) to afford a viscous light yellow liquid **2** (0.87 g, 2.2 mmol, yield: 61%). ^{1}H NMR (400 MHz, $CDCl_3$) δ ppm 7.37 – 7.18 (m, 10H), 5.15 (d, *J* = 3.0 Hz, 1H), 4.88 – 4.74 (m, 2H), 4.60 (d, *J* = 12.0 Hz, 1H), 4.49 (dd, *J* = 11.5, 5.8 Hz, 2H), 4.19 (dd, *J* = 11.5, 3.0 Hz, 1H), 4.03 (dd, *J* = 9.0 Hz, *J* = 8.5 Hz 1H), 3.81 – 3.65 (m, 3H), 3.50 (s, 3H). ^{13}C NMR (101 MHz, $CDCl_3$) δ ppm 153.69, 137.85, 137.08, 128.63, 128.60, 128.25, 128.18, 128.02, 127.99, 95.82, 80.39, 77.34, 74.75, 73.73, 73.01, 72.54, 67.64, 56.06. FT-IR(ATR) 3167-2736, 1807, 1482, 1447, 1350, 1307, 1245, 1201, 1122, 1052, 1000, 921, 833, 719, 684 cm^{-1}. HRMS (ESI$^+$) Calcd. for ($M+H^+$) $C_{12}H_{20}O_7H^+$ 375.1802; found 375.1798.

Synthesis of methyl 4,6-di-*O*-alkylcarbonyl-2,3-*O*-carbonyl-α-D-glucopyranoside (M(RC^2)GC):

Methyl 2,3-*O*-carbonyl-α-D-glucopyranoside, *12*:

To a solution of Methyl 4,6-bezylidene-2,3-*O*-carbonyl-α-D-glucopyranoside (***MBGC***, 3.98 g, 12.9 mmol) in DCM/MeOH (100 mL, v/v=1:1) in a round bottom flask charged with a stir bar, Pd/C (0.402 g, 10 wt% of ***MBGC***) was added. The flask was vacuumed and refilled with N_2 three times and finished at a vacuumized state. A balloon filled with H_2 was connected to the vacuumed flask. The reaction was monitored by TLC and quenched by exposure to the atmosphere after 16 h. Pd/C in the reaction mixture was removed through a silica plug, and the solvent was removed under reduced pressure. The residue was purified by column chromatography (MeOH/DCM= 2:98) to yield a white solid, ***12*** (2.52 g, 11.5 mmol yield 89%). ^{1}H NMR (400 MHz, $CDCl_3$) δ ppm 5.14 (d, *J* = 3.0 Hz, 1H), 4.75 (dd, *J* = 11.5, 10.0 Hz, 1H), 4.25 – 4.08 (m, 2H), 3.94 – 3.84 (m, 2H), 3.63 (dt, *J* = 9.0, 4.0 Hz, 1H), 3.53 (s, 3H), 2.90 (d, *J* = 4.0 Hz, 1H), 1.97-1.92(m, 1H). ^{13}C NMR (101

MHz, $CDCl_3$) δ ppm 153.60, 96.18, 79.67, 77.34, 73.97, 69.32, 61.46, 56.29. FT-IR(ATR) 3622-3129, 3070-2842, 1780, 1412, 1245, 1131, 1000, 938, 815, 771, 728, 649 cm^{-1}. HRMS (ESI^+) Calcd. for ($M+H^+$) $C_8H_{12}O_7H^+$ 221.0656; found 221.0652.

Methyl 4,6-di-*O*-ethyloxycarbonyl-2,3-*O*-carbonyl-α-D-glucopyranoside (M(EC^2)GC), 3:

To a solution of ***12*** (1.03 g, 4.67 mmol) and pyridine (1.8 mL, 22 mmol) in 50 mL DCM, ethyl chloroformate (1.0 mL, 7.1 mmol) in 5 mL DCM was added dropwise. After 1h, diethyl decarbonate (1.6 mL, 1.1 mmol) in 5 mL DCM was added. The reaction was monitored by TLC and was quenched with 5% HCl after 20 h. The organic layer was extracted and washed with brine and dried over Na_2SO_4. The filtered crude was concentrated under reduced pressure, and the residue was purified by column chromatography (EA/Hex=1:4) to yield a viscous transparent colorless liquid (1.40 g, 3.85 mmol, 82%), **3**. ^{1}H NMR (400 MHz, $CDCl_3$) δ ppm 5.24 – 5.11 (m, 2H), 4.83 (dd, *J* = 11.5, 10.0 Hz, 1H), 4.41 – 4.13 (m, 7H), 3.92 (dt, *J* = 9.5, 3.5 Hz, 1H), 3.54 (s, 3H), 1.32 (q, *J* = 7.0 Hz, 6H). ^{13}C NMR (101 MHz, $CDCl_3$) 154.94, 153.57, 152.73, 95.85, 76.80, 76.72, 71.87, 69.84, 65.46, 64.70, 56.50, 14.34, 14.23. FT-IR(ATR) 3030-2789, 1824, 1745, 1447, 1368, 1237, 1157, 1070, 1000, 938, 868, 780, 675 cm^{-1}. HRMS (ESI^+) Calcd. for ($M+Na^+$) $C_{14}H_{20}O_{11}Na^+$ 387.0898; found 387.0891.

Methyl 4,6-di-*O*-*tert* butyloxycarbonyl-2,3-*O*-carbonyl-α-D-glucopyranoside (M(*t*BuC^2)GC), 4:

To a solution of ***12*** (0.98 g, 4.4 mmol) and pyridine (1.9 mL, 23 mmol) in 50 mL DCM, *tert*-butyl decarbonate (3.02 g, 13.8 mmol) in 10 mL DCM was added dropwise. The reaction was monitored by TLC, until the starting material spot disappeared, it was quenched by HCl. The organic layer was washed with brine and dried over Na_2SO_4. The filtered crude was concentrated under reduced pressure and was purified with column chromatography (EA/Hex=1:9) to yield a white solid **4**. (1.71 g, 4.07 mmol, 93%). ^{1}H NMR (500 MHz, $CDCl_3$) δ ppm 5.16 (d, *J* = 3.0 Hz, 1H), 5.11 (dd, *J* = 10.0 Hz, *J* = 9.5 Hz, 1H), 4.81 (dd, *J* = 11.5, 10.0 Hz, 1H), 4.34 (dd, *J* = 12.0, 4.5 Hz, 1H), 4.26 (dd, *J* = 11.5, 3.0 Hz, 1H), 4.15 (dd, *J* = 12.0, 2.5 Hz, 1H), 3.89 (ddd, *J* = 9.5, 4.5, 2.5 Hz, 1H), 3.52 (s, 3H), 1.50 (s, 9H), 1.48 (s, 9H). ^{13}C NMR (126 MHz, $CDCl_3$) δ 153.23, 152.91, 151.63, 95.81, 84.27, 82.89, 77.41, 77.16, 76.91, 76.84, 71.00, 70.12, 63.82, 56.37, 27.86, 27.75. FT-IR(ATR) 3122-2771, 1824, 1745, 1456, 1368, 1254, 1140, 1078, 1000, 938, 850, 780, 675 cm^{-1}. HRMS (ESI^+) Calcd. for ($M+Na^+$) $C_{18}H_{28}O_{11}Na^+$ 443.1524; found 443.1518.

Synthesis of Methyl 4,6-Di-*O*-urethane-2,3-*O*-carbonyl-α-D-glucopyranoside (M(RU^2)GC):

Methyl 4,6-Di-*O*-tosylurethane-2,3-*O*-carbonyl-α-D-glucopyranoside (M(TsU^2)GC), 5:

To a solution of ***12*** (0.50 g, 2.3 mmol) in 10 mL DCM, *p*-toluenesulfonyl isocyanate (0.9 mL, 5.9 mmol) in 5 mL DCM was added dropwise. The reaction was monitored by TLC. Until the starting material spot disappeared; it was quenched by water. The organic layer

was washed with brine and dried over Na_2SO_4. The filtered crude was concentrated under reduced pressure and was purified with column chromatography (EA/DCM=1:9) to yield a white solid **5**. (1.11 g, 1.81 mmol, 79%). ^{1}H NMR (500 MHz, $CDCl_3$) δ 8.18 (d, *J* = 25.0 Hz, 2H), 7.91 (m, 4H), 7.97 – 7.83 (m, 4H), 5.14 (dd, *J* = 10.0 Hz, *J* = 10.0 Hz, 1H), 5.07 (d, *J* = 3.0 Hz, 1H), 4.60 (dd, *J* = 11.5, 10.0 Hz, 1H), 4.30 (dd, J = 12.0, 3.0 Hz, 1H), 4.17 (dd, *J* = 3.0, *J* = 11.5, 1H), 4.05 (dd, *J* = 12.0, 4.0 Hz, 1H), 3.79 (ddd, *J* = 10.0, 4.0, 3.0 Hz, 1H), 3.48 (s, 3H), 2.46 (s, 3H), 2.44 (s, 3H). ^{13}C NMR (126 MHz, $CDCl_3$) δ 152.63, 150.13, 149.05, 146.06, 145.44, 135.54, 135.06, 130.07, 129.88, 128.55, 128.39, 95.78, 76.57, 76.01, 71.18, 69.28, 63.56, 56.61, 21.87. FT-IR(ATR) 3375-2771, 2328, 1820, 1748, 1578, 1443, 1348, 1285, 1209, 1154, 1078, 1008, 938, 863, 818, 762, 661, 583 cm^{-1}. HRMS (ESI$^+$) Calcd. for (M+Na$^+$) $C_{24}H_{26}N_2O_{13}S_2Na^+$ 637.0769; found 637.0757.

General procedure for the organobase-catalyzed ring-opening polymerization of five-membered glucose carbonates

Monomer (100 mg, at predetermined equivalences) was dissolved in ca. 0.4 mL DCM in a vial charged with a stir bar in the glovebox under the Ar atmosphere. A solution of MBA (1 eq.) in 0.05 mL DCM was added, and the content was stirred vigorously for 2 min, followed by a solution of TBD (2 mol% of monomer) in 0.05 mL DCM. The reaction was monitored by SEC with the integration comparison of the polymer peak and monomer peak and quenched by the addition of acetic acid. The polymer was purified by precipitation from DCM into cold methanol three times, followed by drying under a vacuum to afford the polymer as a white solid.

PM(EE^2)GC, 13 ^{1}H NMR (500 MHz, $CDCl_3$) δ 7.15 (AB_q, *J* = 8 Hz, 2H), 5.14 (dd, *J* = 12.1 Hz, *J* = 12.2 Hz), 5.06 – 4.95 (m), 4.53 – 4.38 (m), 3.90 – 3.77 (m), 3.74 – 3.38 (m), 3.30 (s), 2.3 (s, 3H), 1.32 – 0.90 (m). ^{13}C NMR (126 MHz, $CDCl_3$) δ 154.32, 138.60, 132.41, 129.31, 128.77, 96.60, 76.55, 76.48, 74.61, 70.09, 69.01, 68.28, 67.07, 55.20, 15.63, 15.20. FT-IR(ATR) 3024 – 2785, 1759, 1450,1373, 1334, 1273, 1234, 1165, 1095, 1046, 918, 679 cm^{-1}. T_g = 139 °C. TGA in N_2: 220 - 400°C, 95% mass loss. SEC (THF, PS standards): M_n = 12.1 kDa, $Đ$ = 1.11.

PM(BnE^2)GC, 14 ^{1}H NMR (500 MHz, $CDCl_3$) δ 7.46 – 6.96 (m), 5.20 (t, *J* = 9.5 Hz), 5.05 – 4.94 (m), 4.69 – 4.05 (m), 3.85 – 3.18 (m), 3.13 – 2.95 (m), 2.17 (s, 3H). ^{13}C NMR (126 MHz, $CDCl_3$) δ 154.06, 137.81, 128.58, 128.43, 128.08, 127.99, 127.57, 96.44, 76.59, 76.42, 75.05, 74.72, 73.86, 69.90, 68.19, 55.13. FT-IR(ATR) 3008 – 2792, 1759, 1450, 1281, 1234, 1087,1049, 1003, 910, 787, 740, 695 cm^{-1}. T_g = 39 °C. TGA in N_2: 210-400°C, 98% mass loss. SEC (THF, PS standards): M_n = 11.8 kDa, $Đ$ = 1.29.

PM(EC^2)GC, 15 ^{1}H NMR (500 MHz, $CDCl_3$) δ , 7.17 – 7.11 (br), 5.49 – 3.85 (br), 3.47 – 3.33 (br), 2.33 (s, 3H), 1.37 – 1.21 (br). ^{13}C NMR (126 MHz, $CDCl_3$) δ 155.00, 153.99, 96.29, 74.46, 72.35, 66.89, 65.11, 64.49, 55.77, 14.33, 14.25. FT-IR(ATR) 3032 – 2816,

1751, 1458, 1373, 1242, 1165, 1033, 1002, 871, 786 cm^{-1}. T_g = °C. TGA in N_2: 240-400°C, 92% mass loss. SEC (THF, PS standards): M_n = 11.4 kDa, $Đ$ = 1.11.

PM(*t*BuC²)GC, 16 ^{1}H NMR (500 MHz, $CDCl_3$) δ 7.13 (AB_q, J = 8 Hz, 2H), 5.42 – 3.83 (br), 3.59 – 3.19 (br), 2.33 (s, 3H), 1.73 – 1.09 (). ^{13}C NMR (126 MHz, $CDCl_3$) δ 153.83, 153.29, 151.90, 96.42, 83.94, 82.54, 74.50, 74.06, 70.90, 67.16, 64.27, 56.48, 27.91, 27.81. FT-IR(ATR) 3024 – 2823, 1751, 1458, 1365, 1250, 1157, 1103, 1041, 856, 779 cm^{-1}. TGA in N_2: 160 – 200 °C, 48 % mass loss, 230 – 380 °C, 49 % mass loss. SEC (THF, PS standards): M_n = 12.0 kDa, $Đ$ =1.21.

Oligomerization of each monomer (1 – 4*)* and isolation of their corresponding unimers and dimers

The acetal protected glucose monomers (1 eq.) (**1 – 4)** and 4-methylbenzyl alcohol (2 eq.) were dissolved in *ca.* 0.5 mL anhydrous DCM in a vial charged with a stir bar in the glovebox under Ar atmosphere. After fitting the vial with a septum, the reaction mixture was transferred to a fume hood equipped with a Schlenk line. The reaction mixture was stirred vigorously for 2 min, and a solution of TBD (0.02 eq.) in 0.1 mL anhydrous DCM was added. After 1.5 h, the reaction vial was opened to air and quenched by the addition of acetic acid. The unimers were purified and fractionated using preparative SEC for up to 3 cycles. The two regioisomeric unimers were then separated by column chromatography (ethyl acetate/hexanes) and determined using 1D and 2D NMR spectroscopies.

II. Figures

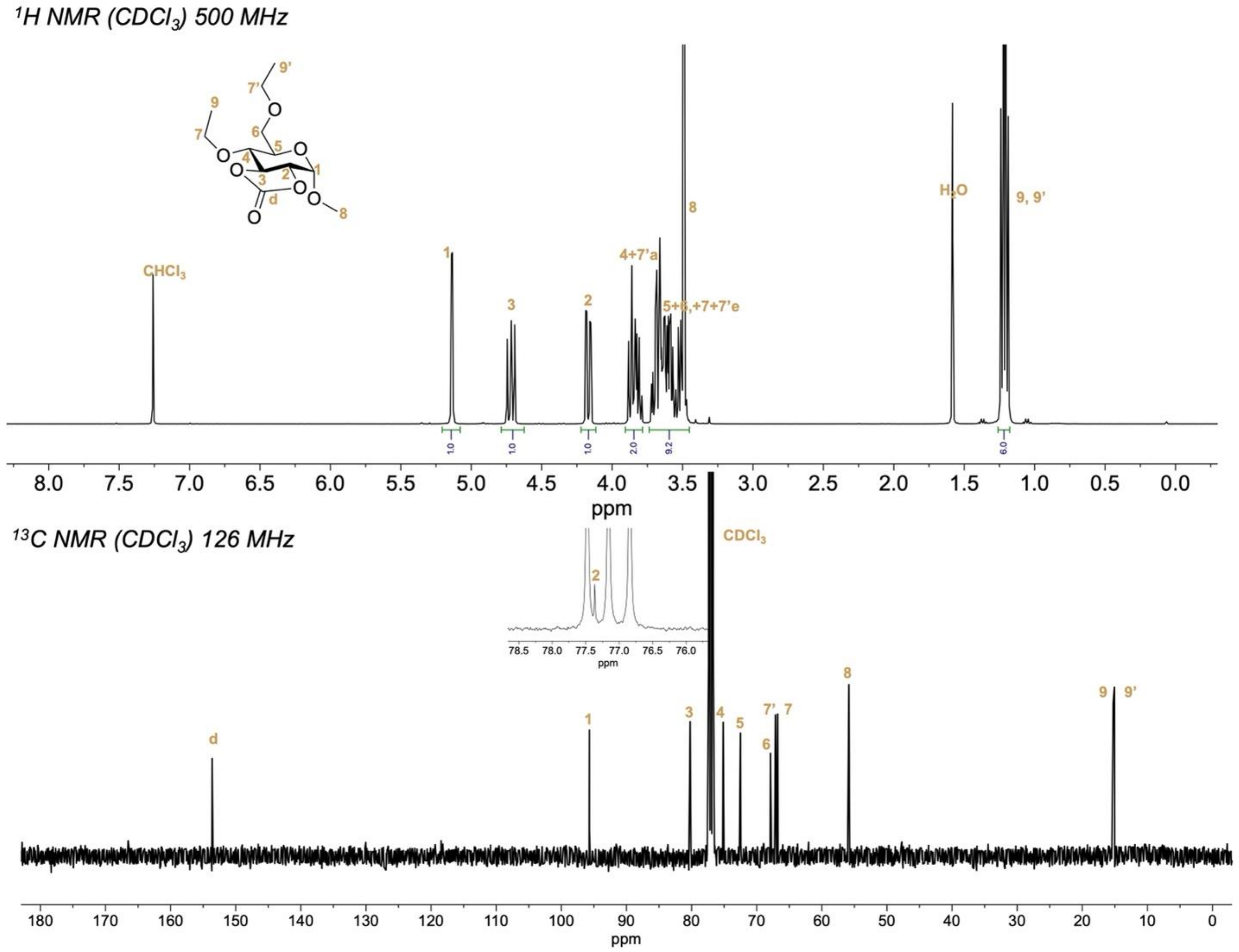

Figure S1. ^{1}H NMR (500 MHz) and ^{13}C NMR (126 MHz) spectra of M(EE^2)GC (**1**) in $CDCl_3$.

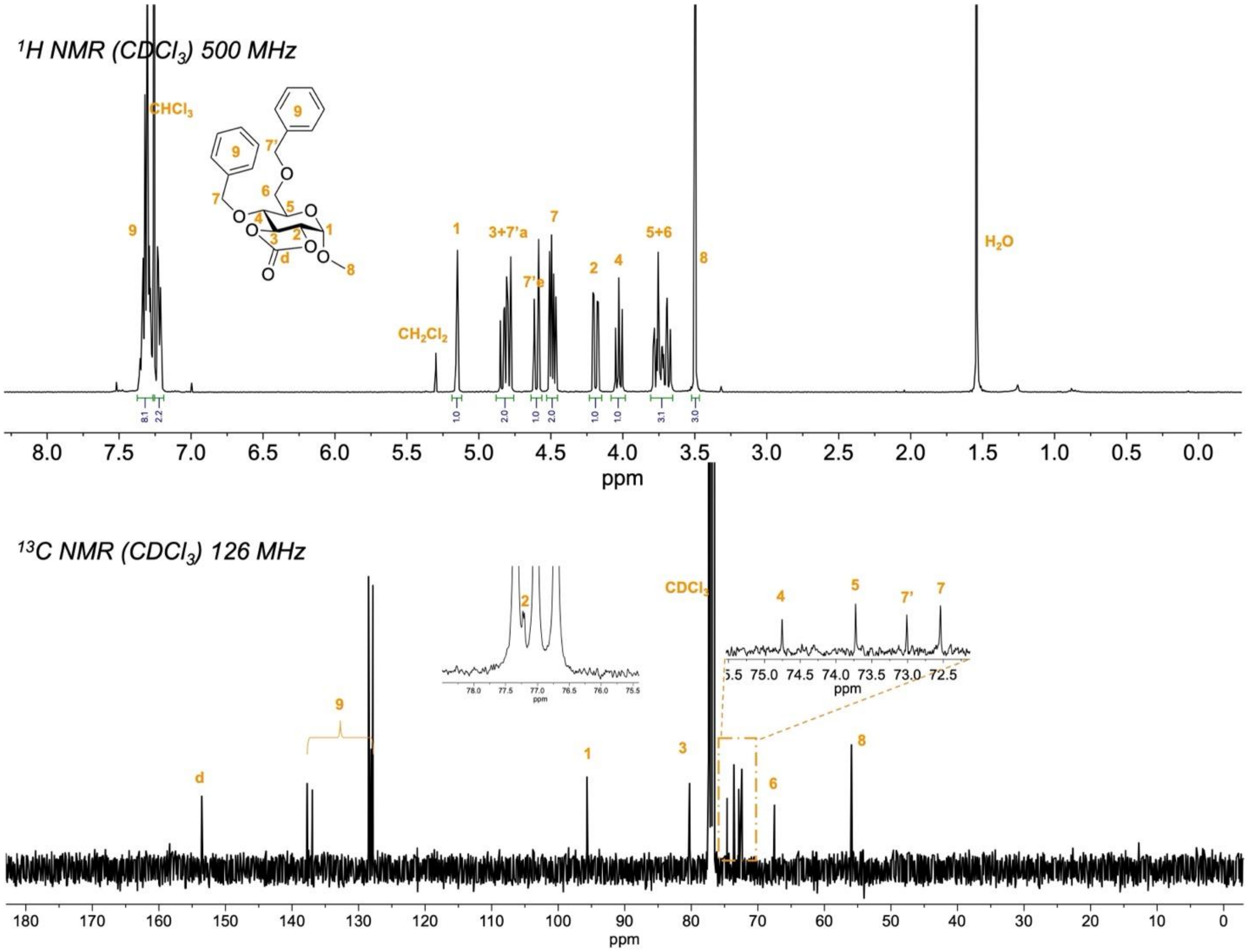

Figure S2. ^{1}H NMR (500 MHz) and ^{13}C NMR (126 MHz) spectra of M(BnE^{2})GC (**2**) in $CDCl_3$.

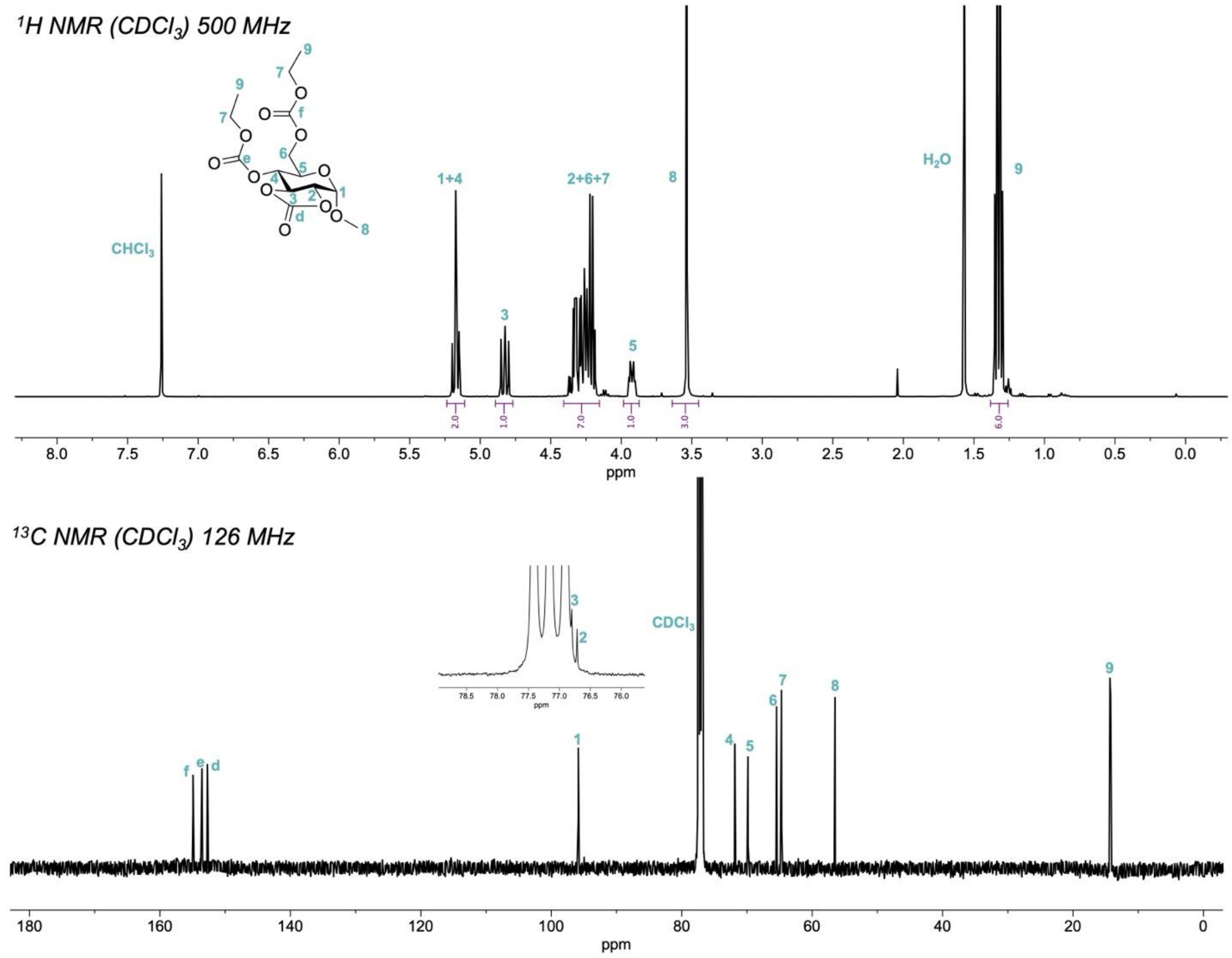

Figure S3. ^{1}H NMR (500 MHz) and ^{13}C NMR (126 MHz) spectra of M(EC^{2})GC (**3**) in $CDCl_3$.

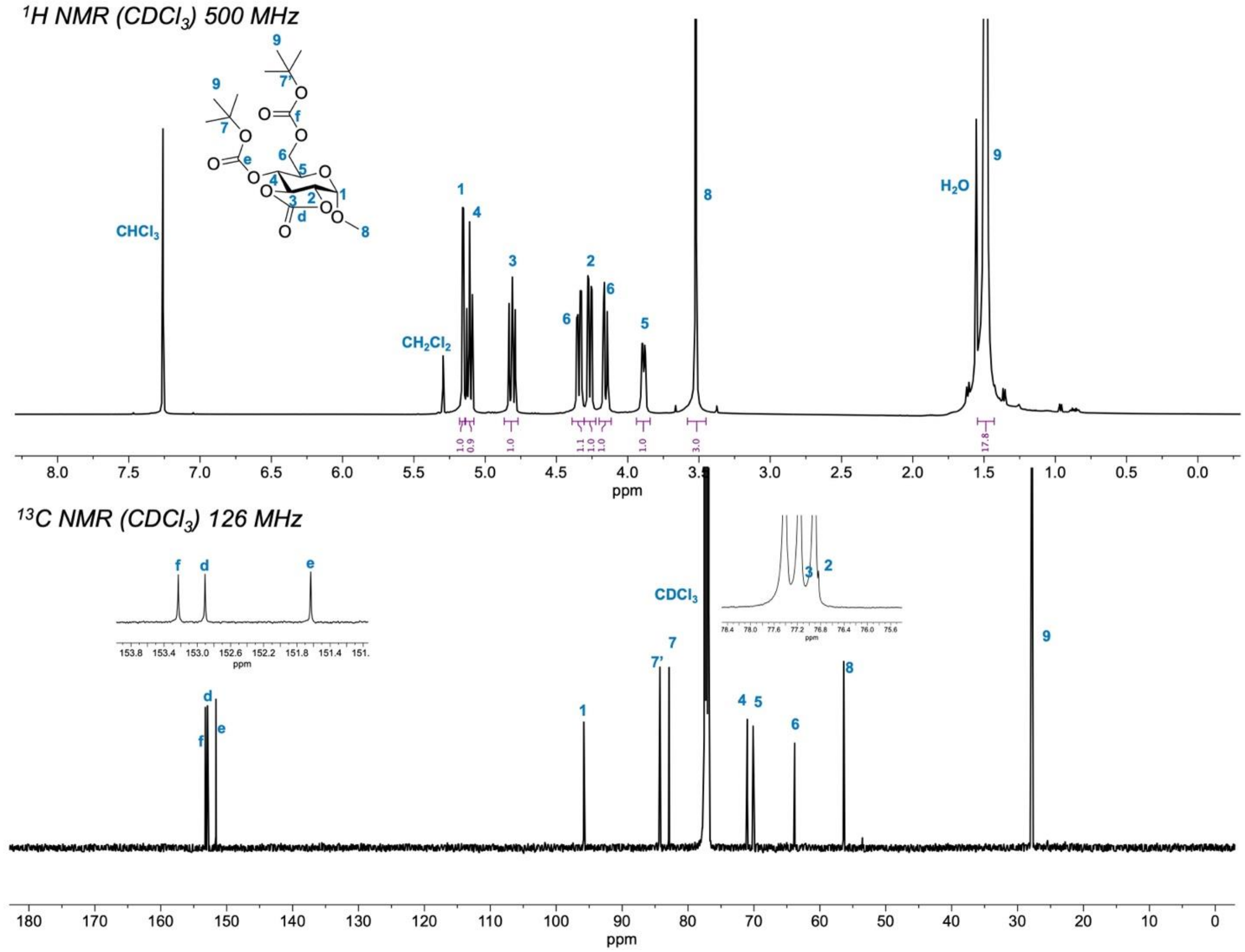

Figure S4. ^{1}H NMR (500 MHz) and ^{13}C NMR (126 MHz) spectra of M(*t*BuC^{2})GC (**4**) in $CDCl_3$.

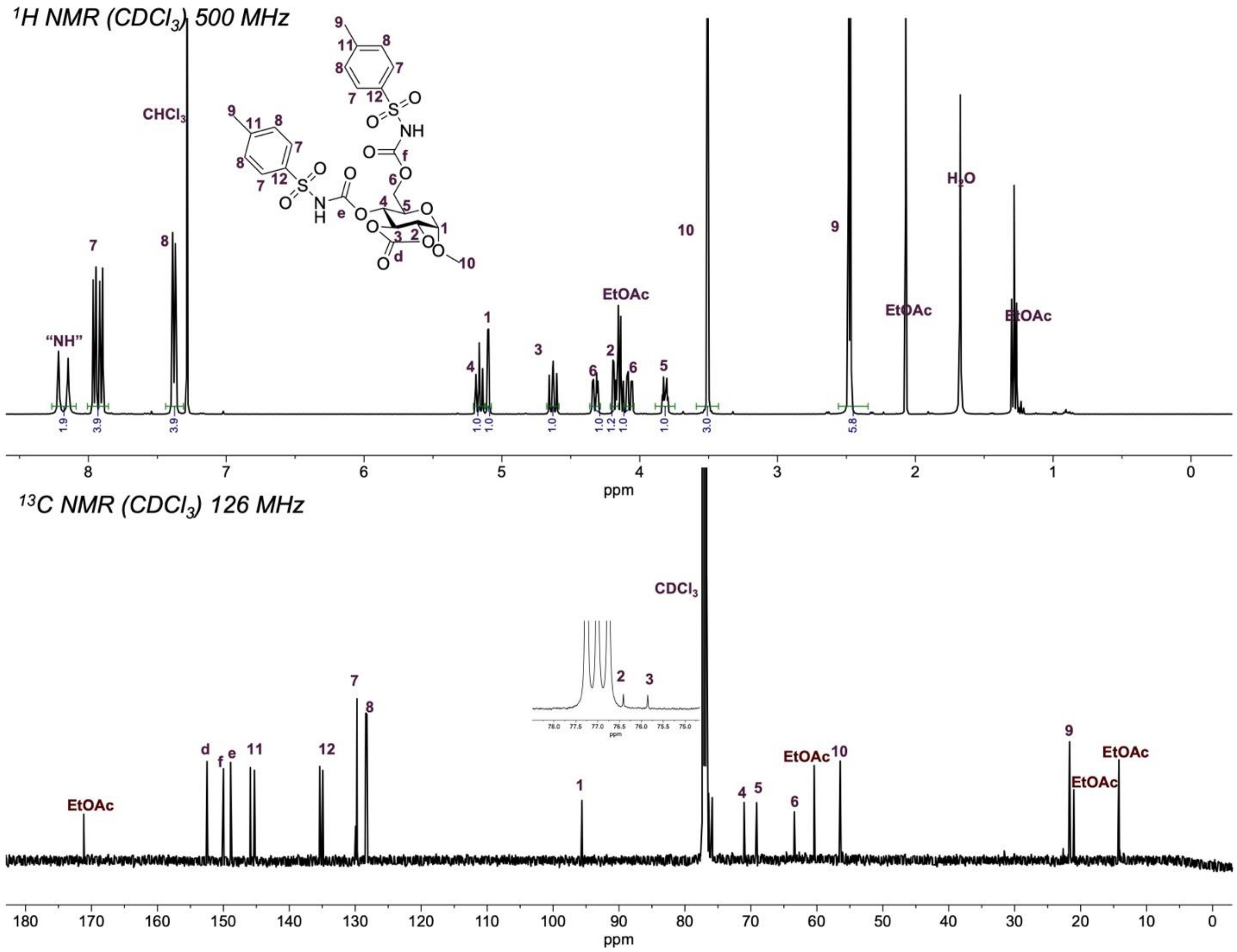

Figure S5. ^{1}H NMR (500 MHz) and ^{13}C NMR (126 MHz) spectra of M(TsU^{2})GC (**5**) in $CDCl_3$.

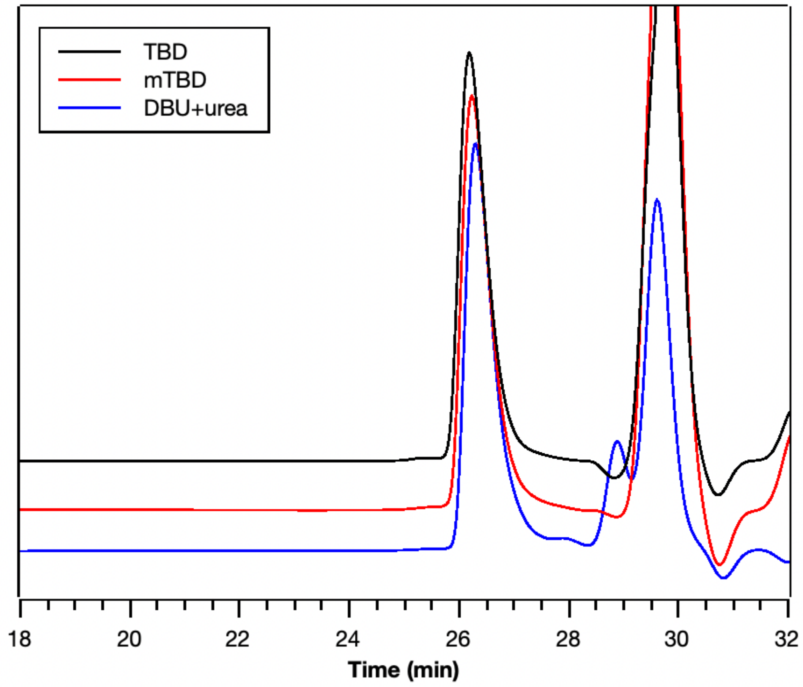

Figure S6. ROPs of M(TsU^2)GC, **5** with different catalysts after 20 h.

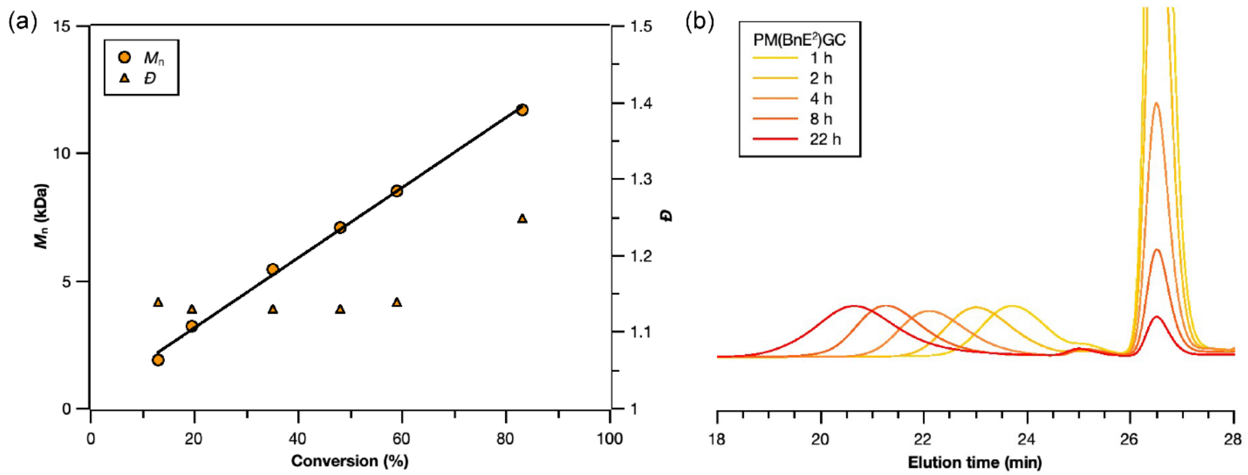

Figure S7. (a) Plot of M_n and $Đ$ as a function of monomer conversion (%) for the polymerization of M(BnE^2)GC, **2** using **TBD** as catalyst. (b) SEC traces (THF as eluent, 1mL/min) of the ROP as a function of polymerization time, with normalization of the intensity of the polymer peaks.

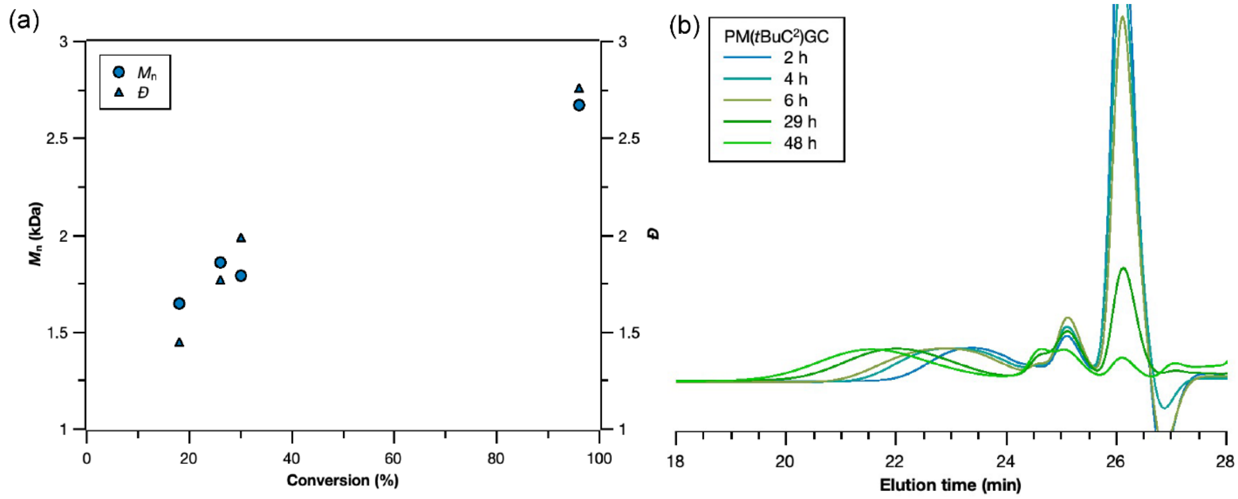

Figure S8. (a) Plot of M_n and $Đ$ as a function of monomer conversion (%) for the polymerization of M(*t*BuC^2)GC, **4** using **TBD** as catalyst. (b) SEC traces (THF as eluent, 1mL/min) of the ROP as a function of polymerization time, with normalization of the intensity of the polymer peaks.

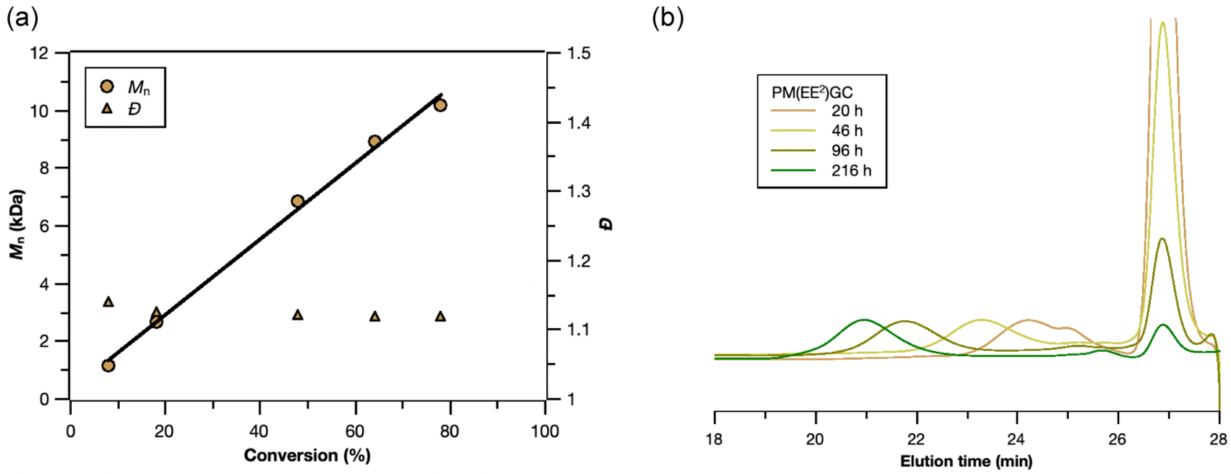

Figure S9. (a) Plot of M_n and $Đ$ as a function of monomer conversion (%) for the polymerization of M(EE^2)GC, **1** using **mTBD** as catalyst. (b) SEC traces (THF as eluent, 1mL/min) of the ROP as a function of polymerization time, with normalization of the intensity of the polymer peaks.

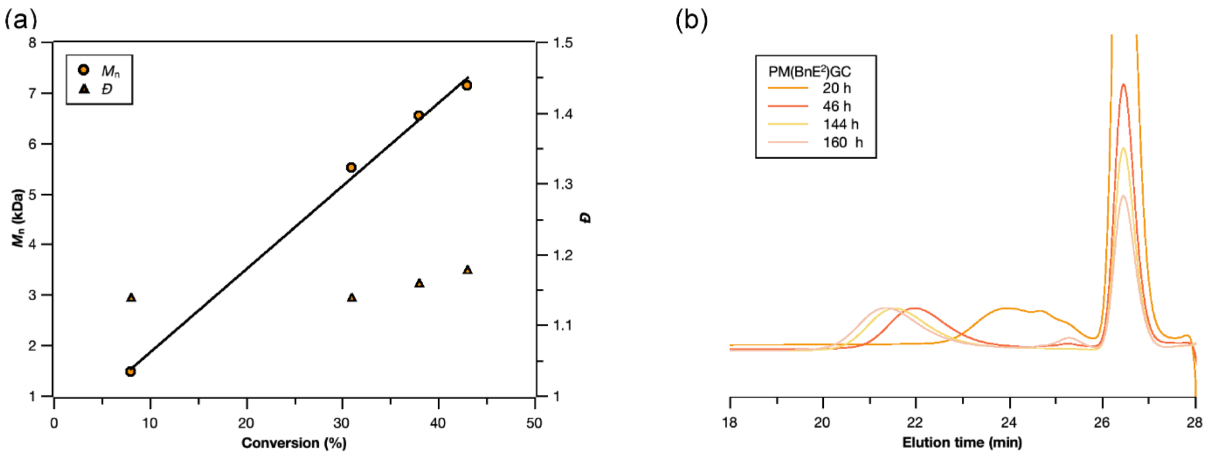

Figure S10. (a) Plot of M_n and $Đ$ as a function of monomer conversion (%) for the polymerization of M(BnE^2)GC, **2** using **mTBD** as catalyst. (b) SEC traces (THF as eluent, 1mL/min) of the ROP as a function of polymerization time, with normalization of the intensity of the polymer peaks.

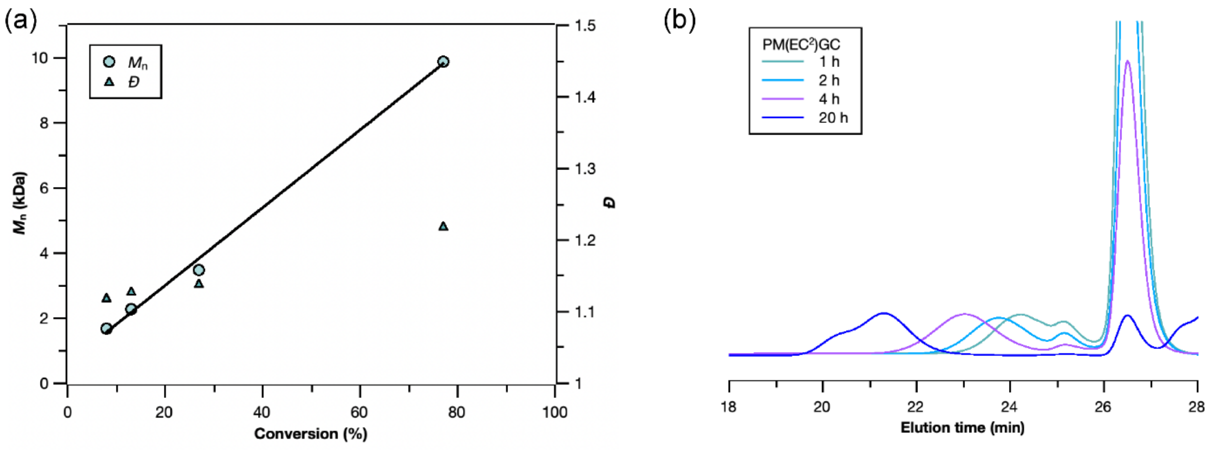

Figure S11. (a) Plot of M_n and $Đ$ as a function of monomer conversion (%) for the polymerization of M(EC^2)GC, **3** using **mTBD** as catalyst. (b) SEC traces (THF as eluent, 1mL/min) of the ROP as a function of polymerization time, with normalization of the intensity of the polymer peaks.

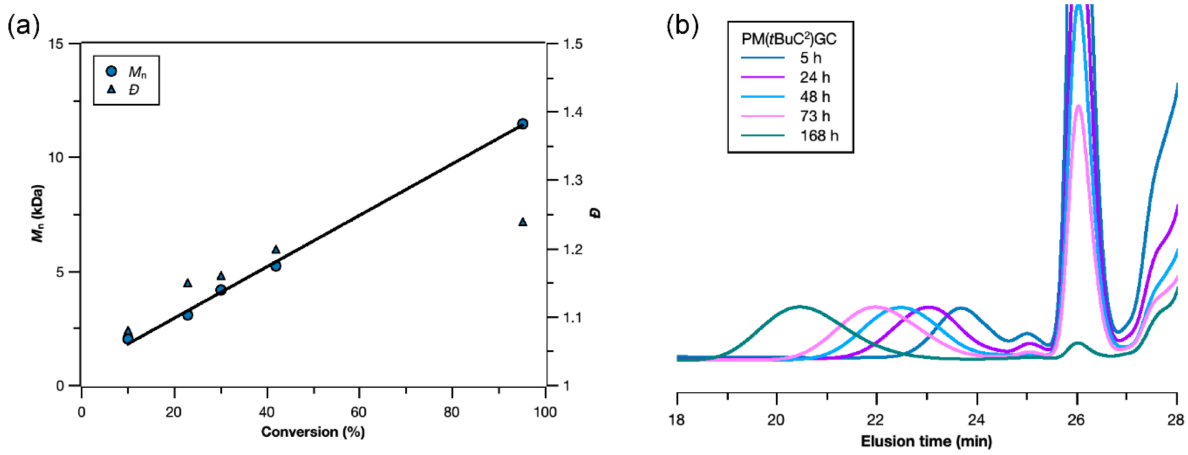

Figure S12. (a) Plot of M_n and $Đ$ as a function of monomer conversion (%) for the polymerization of M(*t*BuC^2)GC, **4** using **mTBD** as catalyst. (b) SEC traces (THF as eluent, 1mL/min) of the ROP as a function of polymerization time, with normalization of the intensity of the polymer peaks.

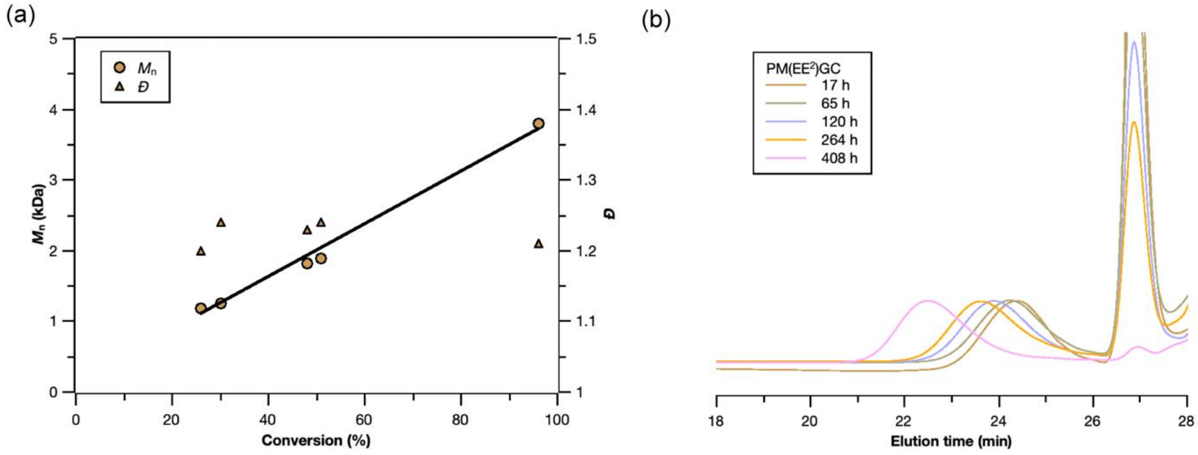

Figure S13. (a) Plot of M_n and $Đ$ as a function of monomer conversion (%) for the polymerization of M(EE^2)GC, **1** using **DBU+Urea** as catalyst. (b) SEC traces (THF as eluent, 1mL/min) of the ROP as a function of polymerization time, with normalization of the intensity of the polymer peaks.

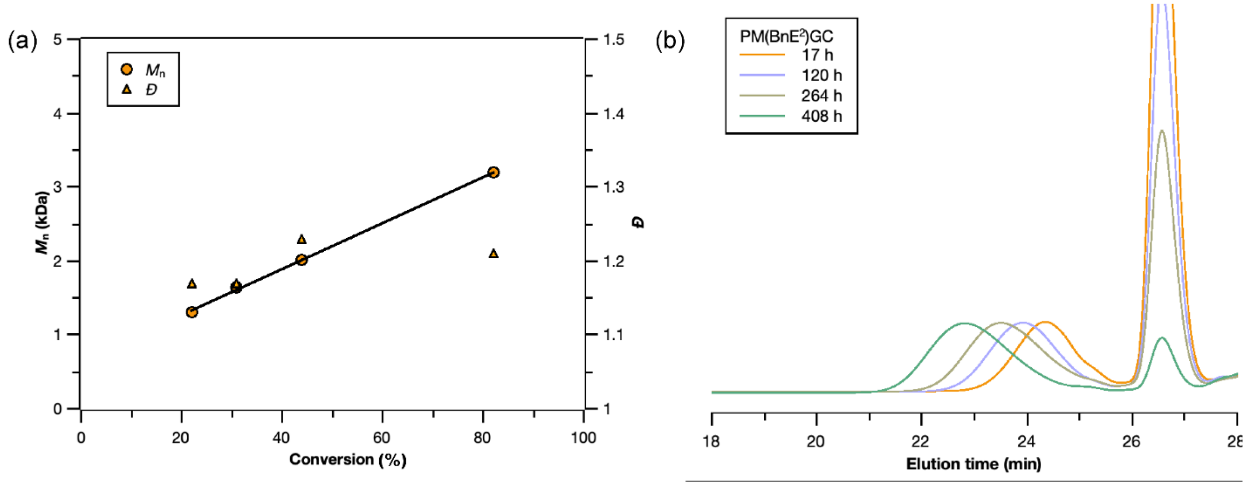

Figure S14. (a) Plot of M_n and *Đ* as a function of monomer conversion (%) for the polymerization of M(BnE^2)GC, **2** using **DBU+Urea** as catalyst. (b) SEC traces (THF as eluent, 1mL/min) of the ROP as a function of polymerization time, with normalization of the intensity of the polymer peaks.

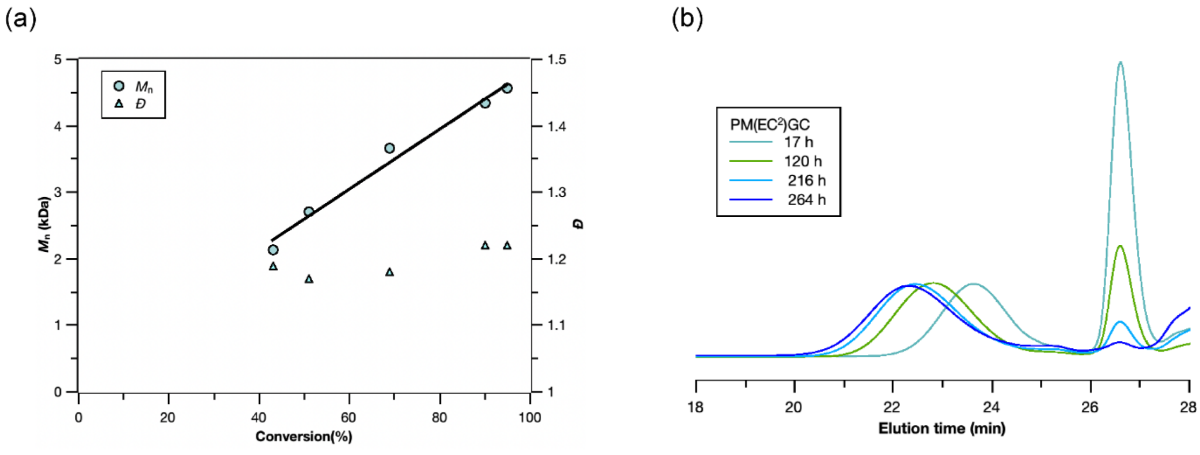

Figure S15. (a) Plot of M_n and *Đ* as a function of monomer conversion (%) for the polymerization of M(EC^2)GC, **3** using **DBU+Urea** as catalyst. (b) SEC traces (THF as eluent, 1mL/min) of the ROP as a function of polymerization time, with normalization of the intensity of the polymer peaks.

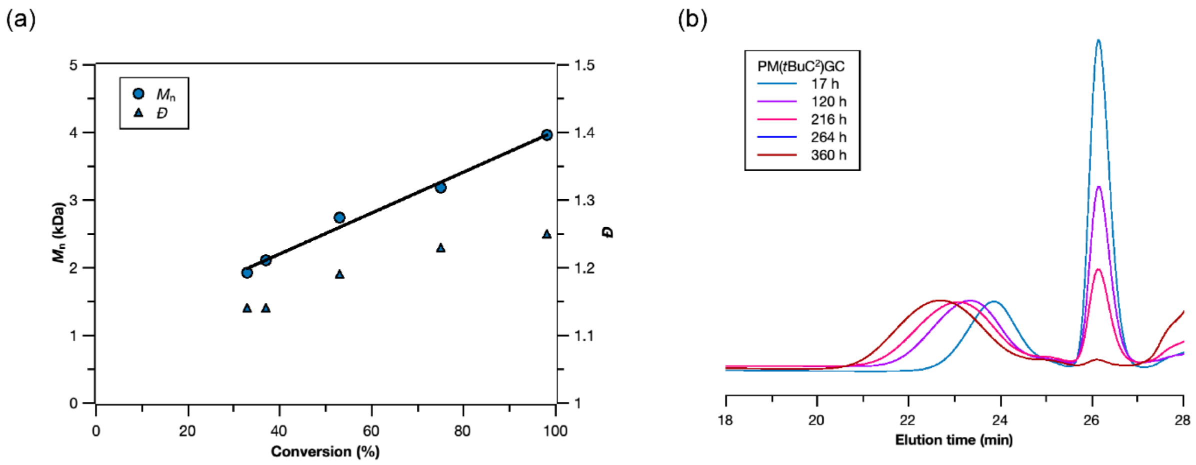

Figure S16. (a) Plot of M_n and $Đ$ as a function of monomer conversion (%) for the polymerization of M(*t*BuC[2])GC, **4** using **DBU+urea** as catalyst. (b) SEC traces (THF as eluent, 1mL/min) of the ROP as a function of polymerization time, with normalization of the intensity of the polymer peaks.

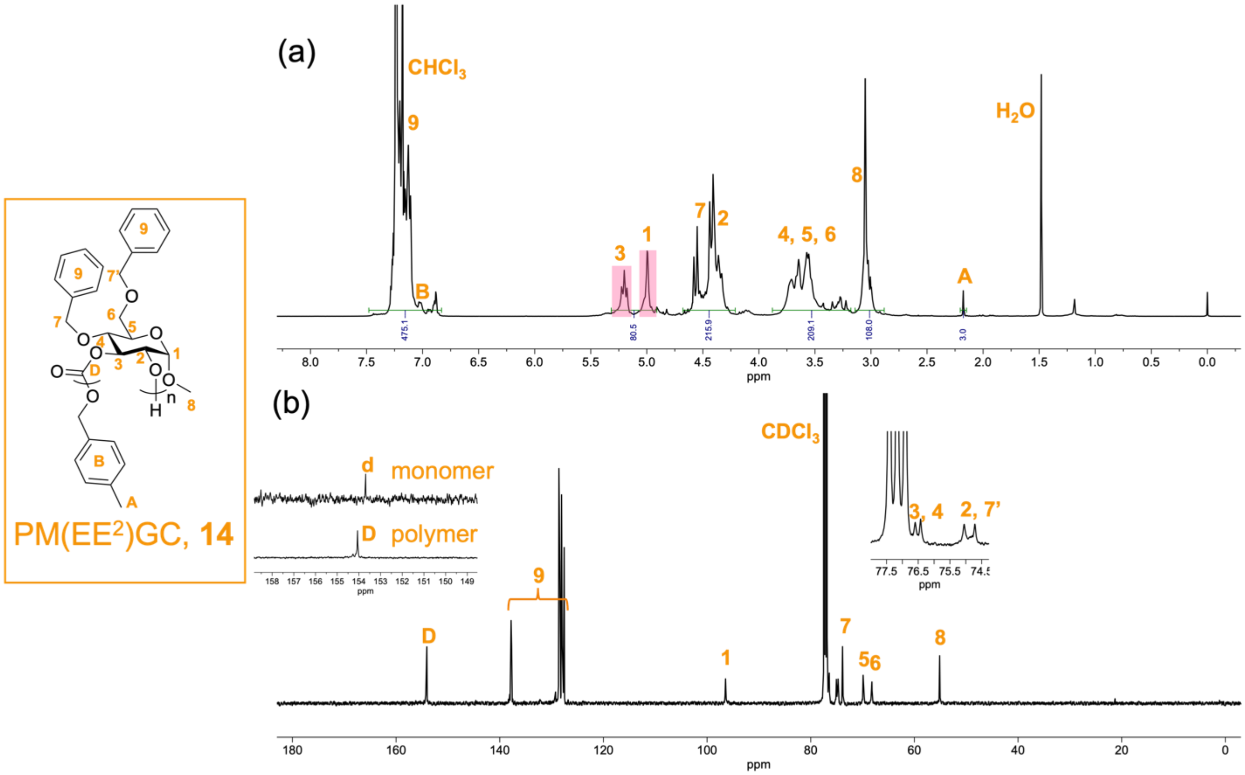

Figure S17. ^1H NMR (500 MHz) and ^{13}C NMR (126 MHz) spectra of PM(BnE[2])GC (**14**) *via* TBD-catalytic ROP in $CDCl_3$.

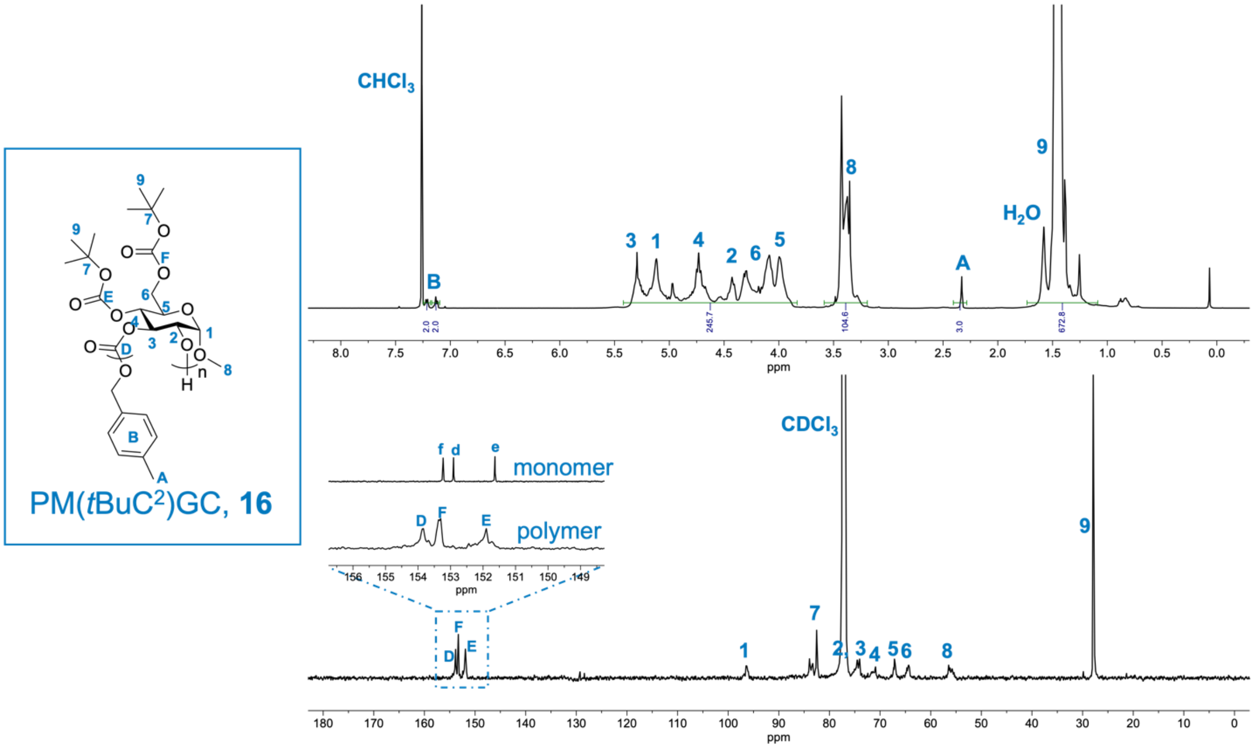

Figure S18. ^{1}H NMR (500 MHz) and ^{13}C NMR (126 MHz) spectra of PM(*t*BuC^{2})GC (**16**) *via* TBD-catalytic ROP in $CDCl_3$.

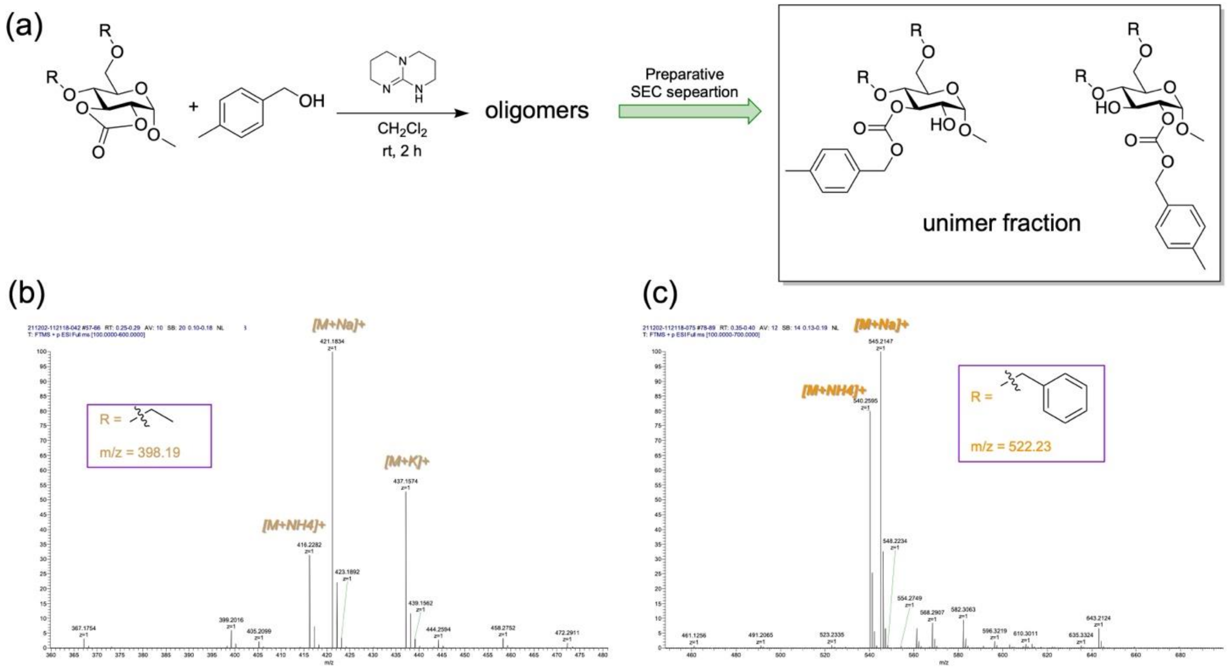

Figure S19. (a) Scheme of unimer preparation, MAlDI-TOF spectrum of unimers of (b) M(EE^{2})GC, ***7*** and (c) M(BnE^{2})GC, ***8***.

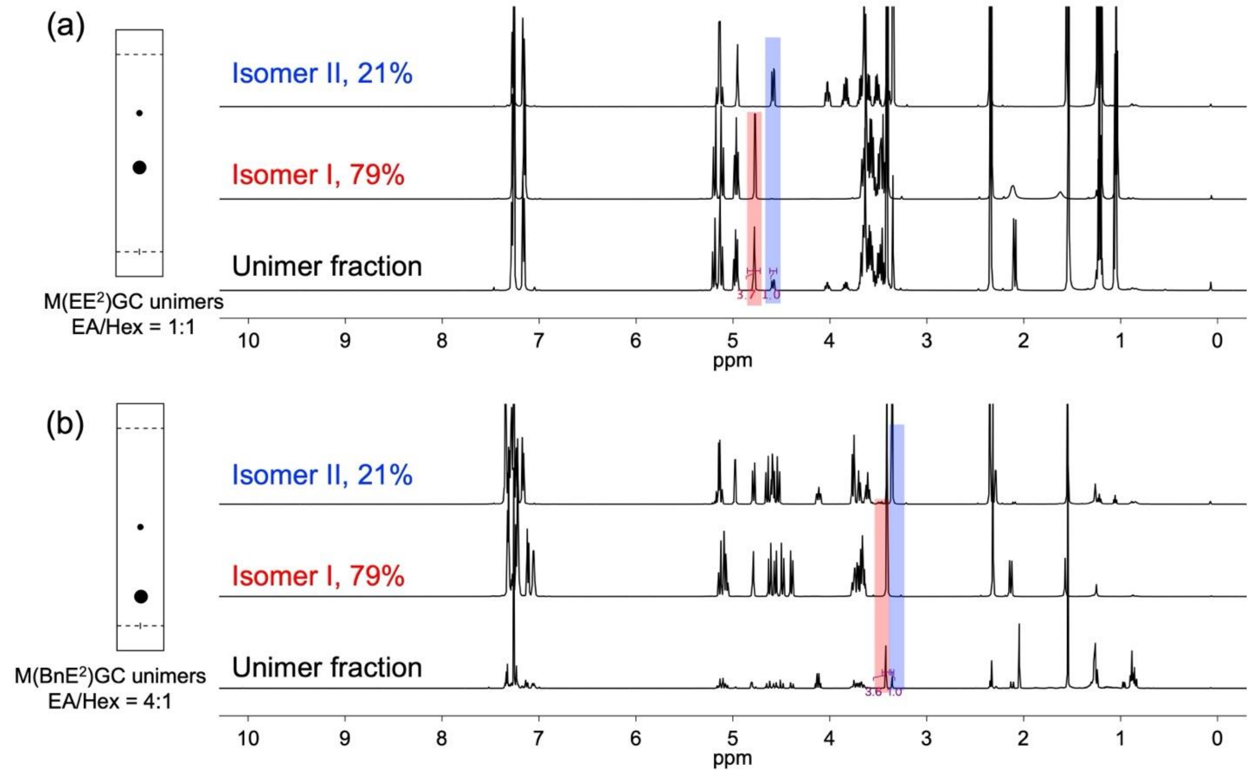

Figure S20. ^{1}H NMR spectrum of a crude mixture of the unimeric isomeric isomers (a) of M(EE^2)GC and (b) of M(BnE^2)GC.

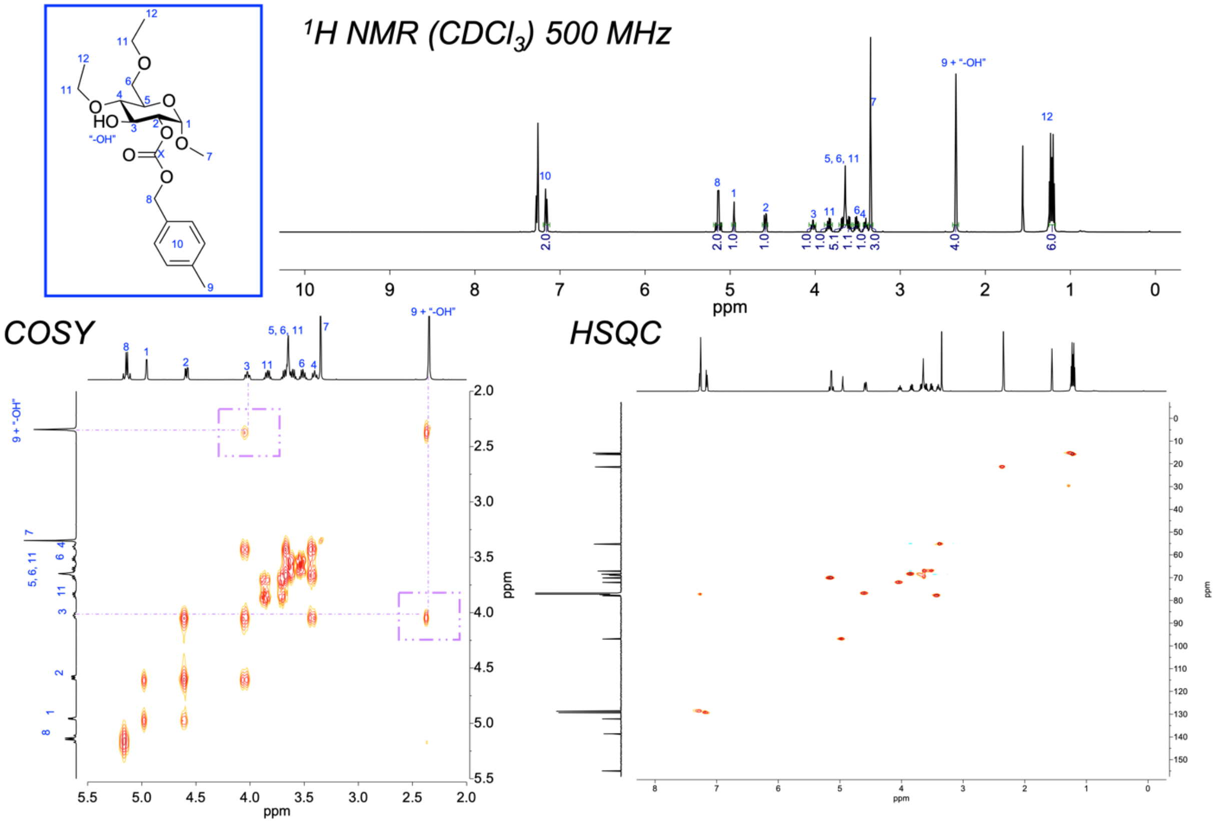

Figure S21. ^{1}H NMR, COSY and HSQC spectrum of M(EE^{2})GC unimer regioisomer II in $CDCl_3$.

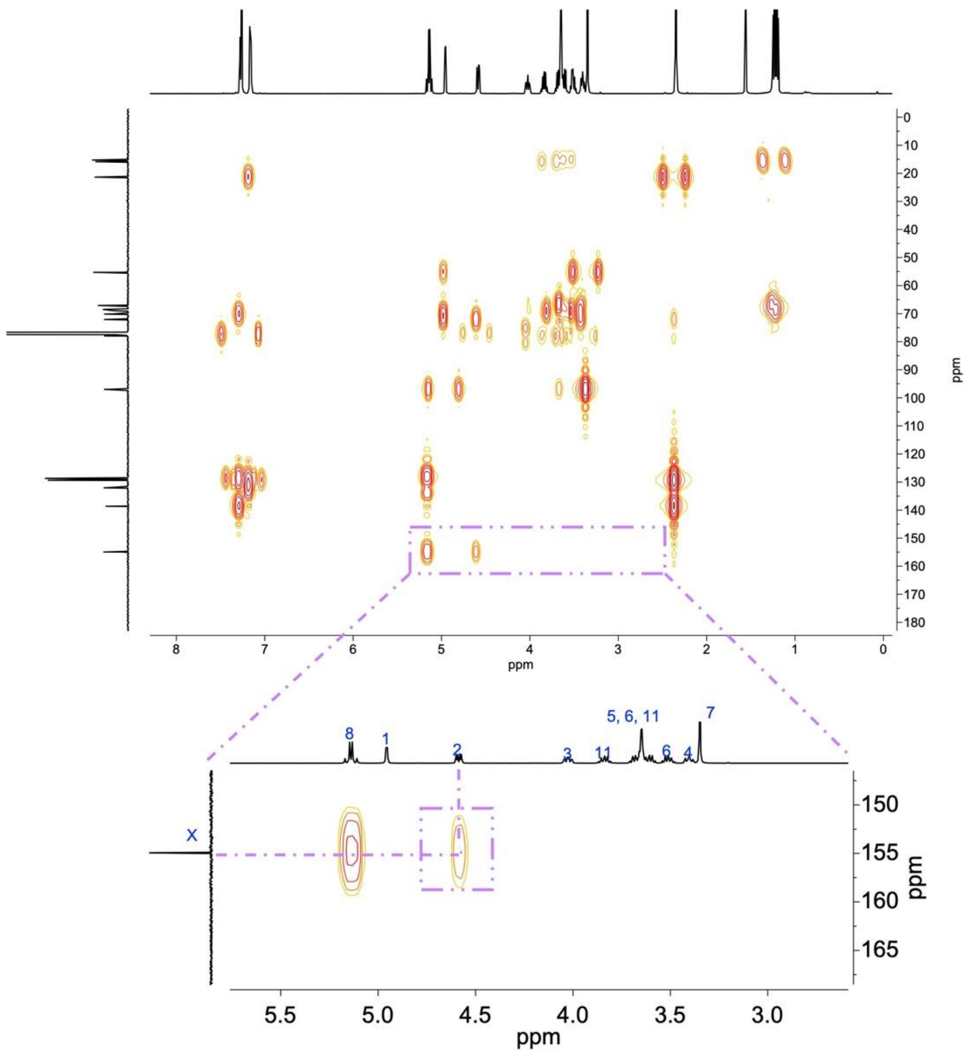

Figure S22. HMBC spectrum of M(EE^2)GC unimer regioisomer II in $CDCl_3$.

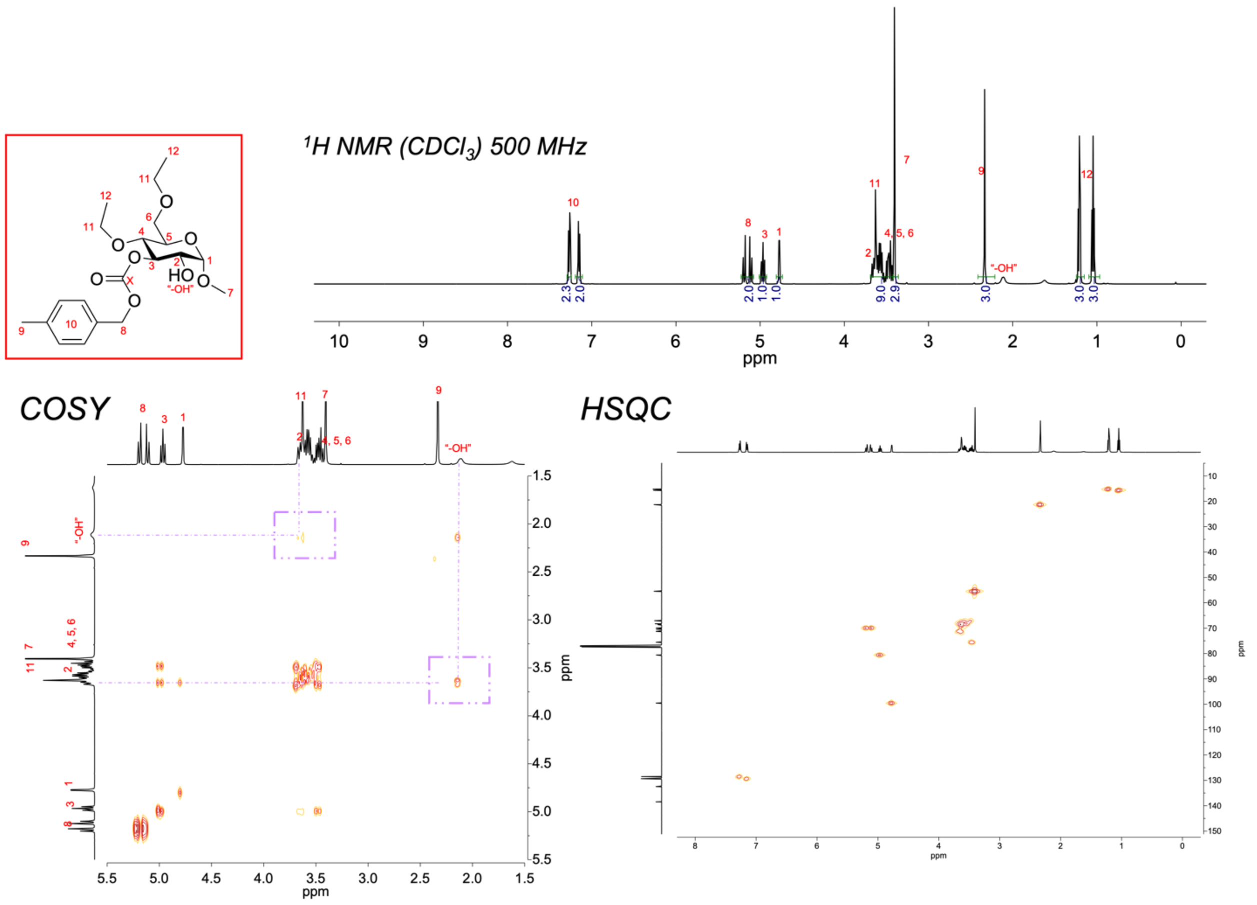

Figure S23. ^{1}H NMR, COSY and HSQC spectrum of M(EE[2])GC unimer regioisomer I in $CDCl_3$.

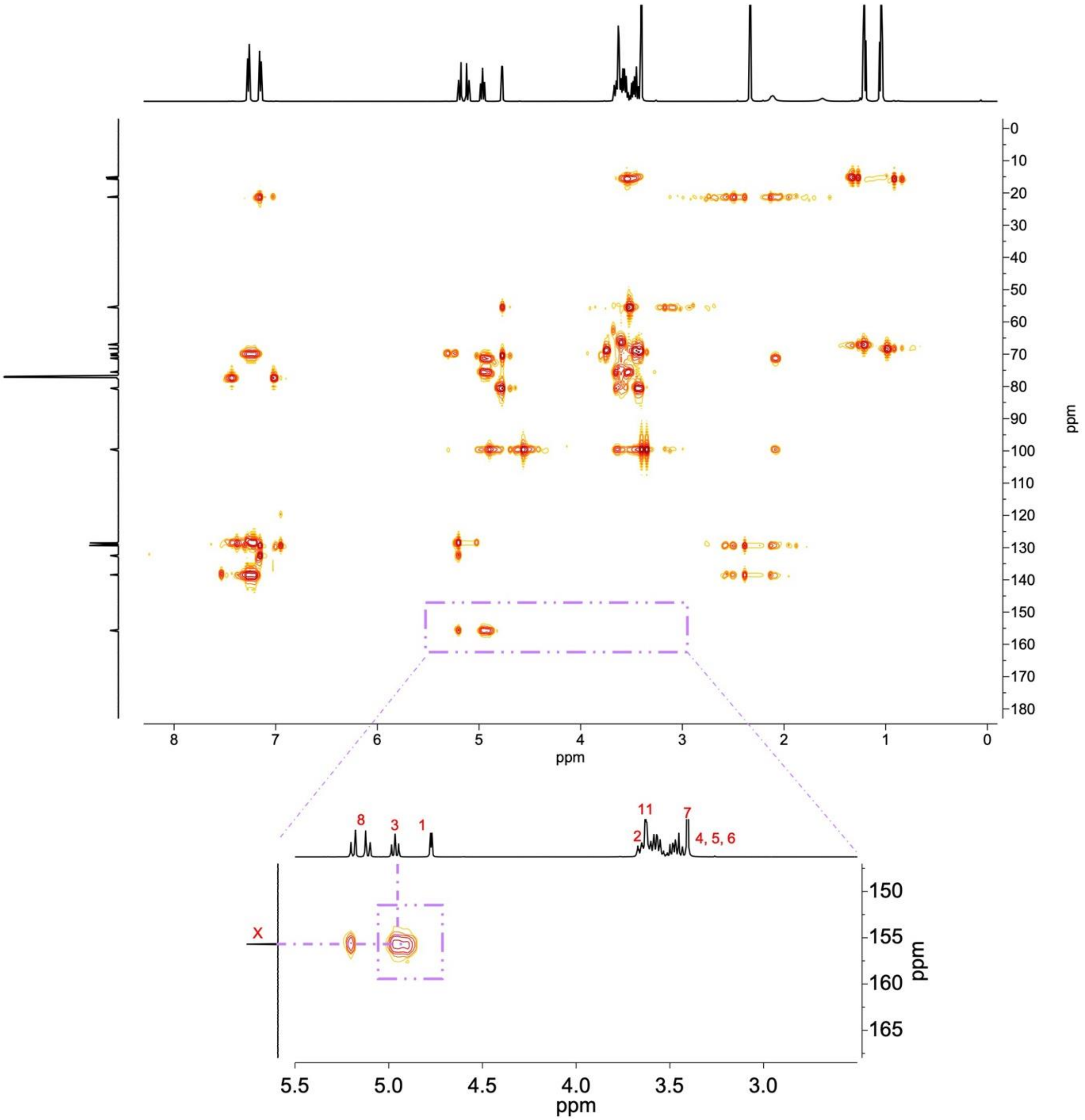

Figure S24. HMBC spectrum of M(EE^2)GC unimer regioisomer I in $CDCl_3$.

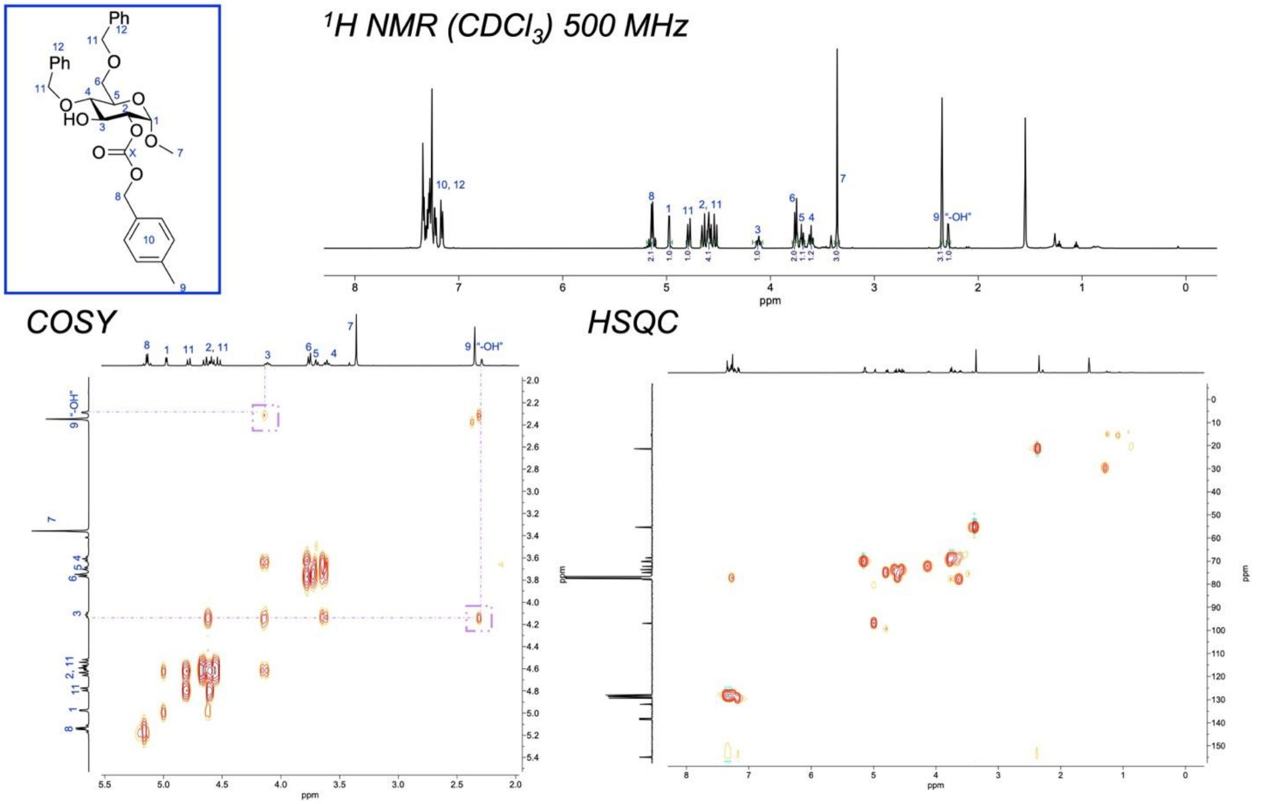

Figure S25. ^{1}H NMR, COSY and HSQC spectrum of M(BnE^{2})GC unimer regioisomer II in $CDCl_3$.

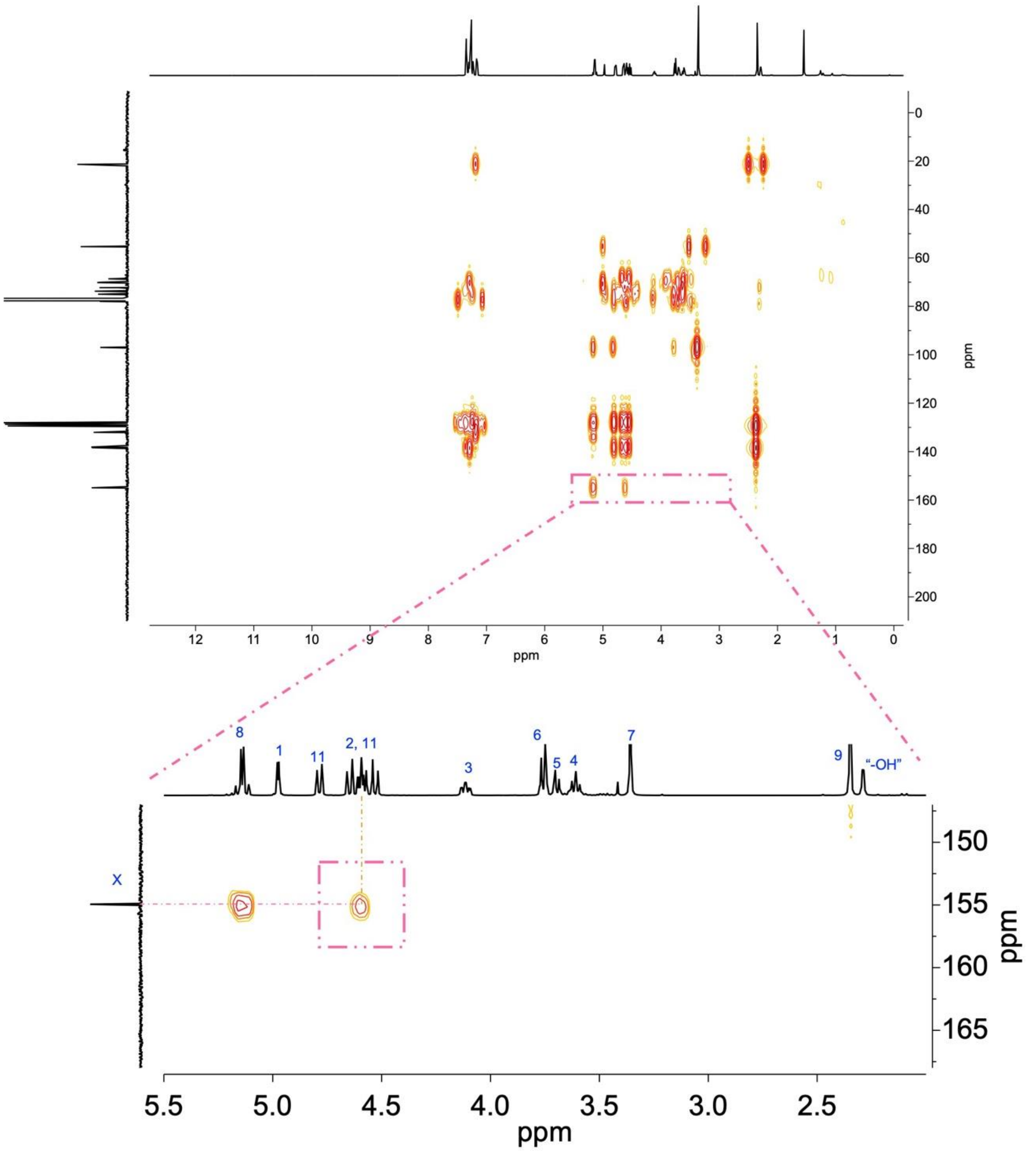

Figure S26. HMBC spectrum of M(BnE[2])GC unimer regioisomer II in $CDCl_3$.

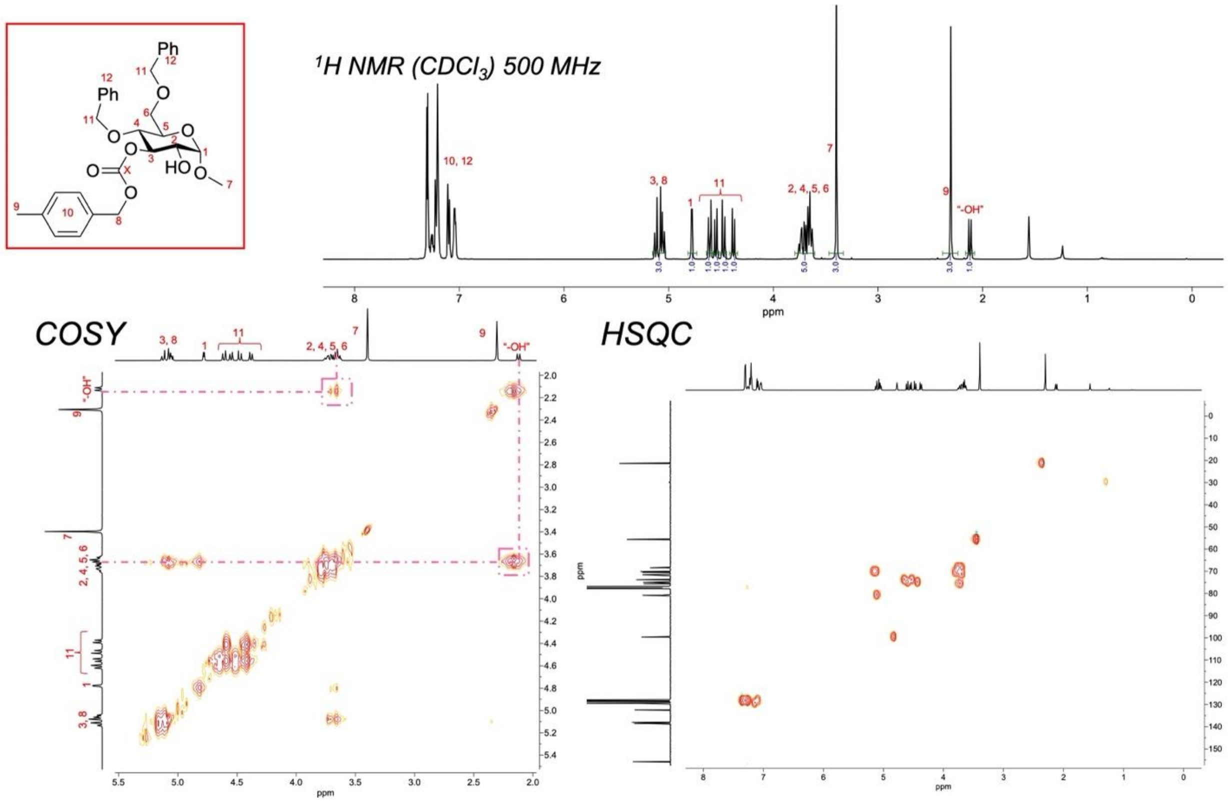

Figure S27. ^{1}H NMR, COSY and HSQC spectrum of M(BnE^2)GC unimer regioisomer I in $CDCl_3$.

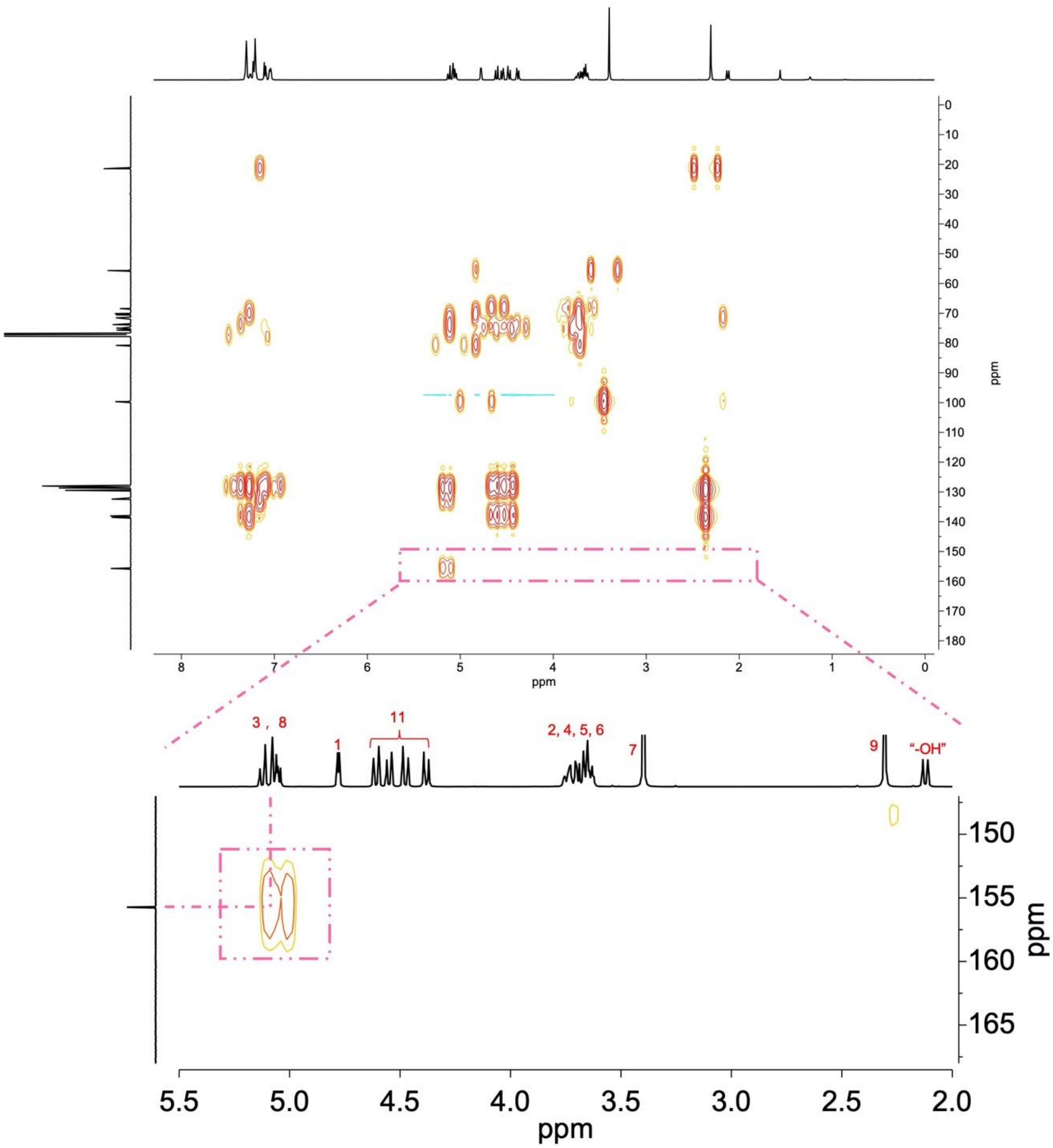

Figure S28. HMBC spectrum of M(EE^2)GC unimer regioisomer I in $CDCl_3$.

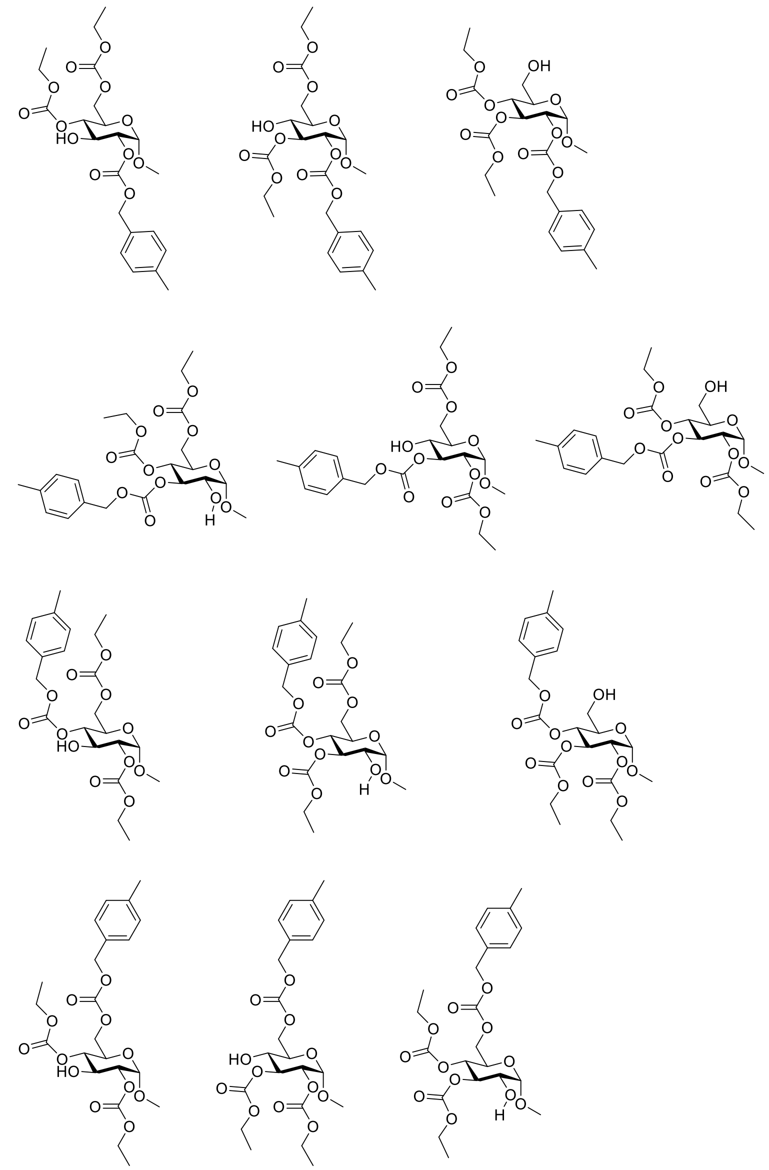

Figure S29. Possible unimer structures of M(EC^2)GC with the occurrences of intra- and intermolecular transcarbonylation reactions.

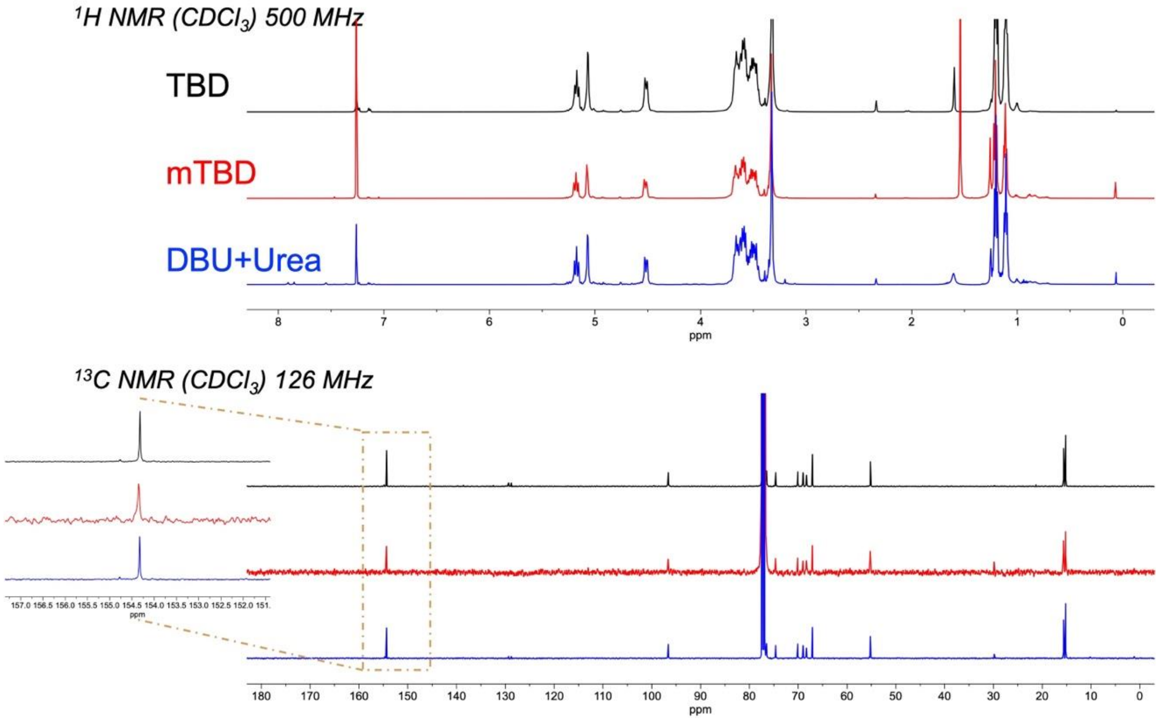

Figure S30. Comparison of ^{1}H NMR (500 MHz) and ^{13}C NMR (126 MHz) spectra of PM(EE^{2})GC (**13**) *via* ROP using different catalyst in $CDCl_3$.

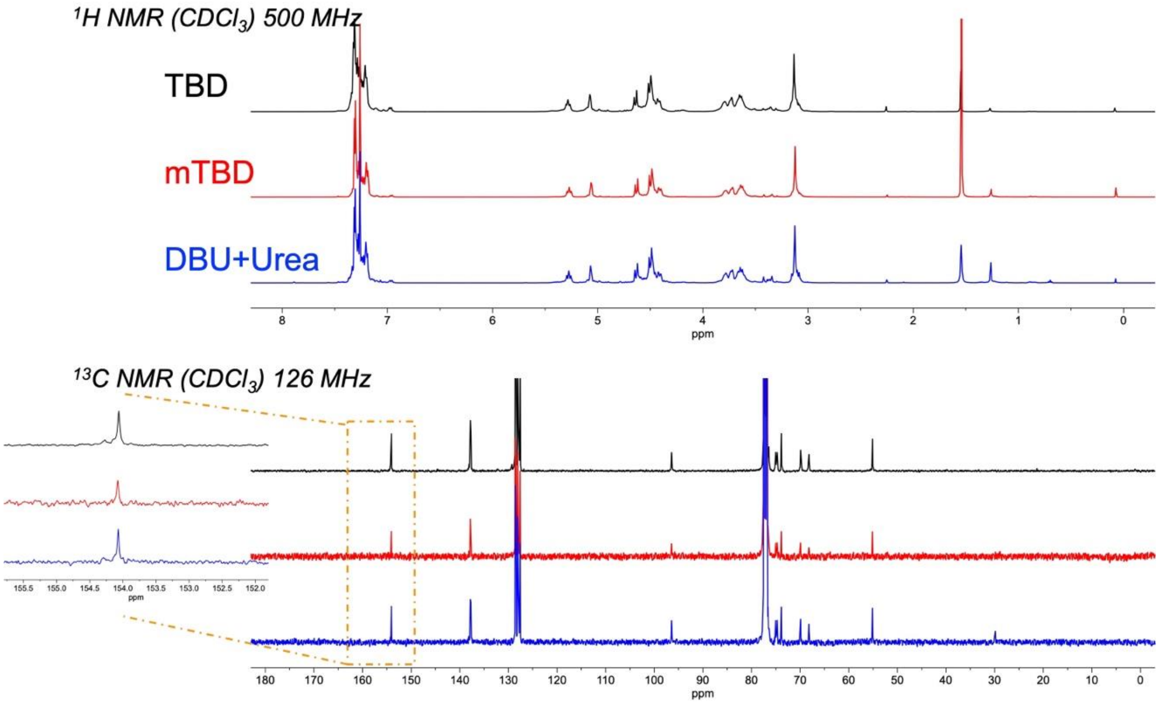

Figure S31. Comparison of ^{1}H NMR (500 MHz) and ^{13}C NMR (126 MHz) spectra of PM(BnE^{2})GC (**14**) *via* ROP using different catalyst in $CDCl_3$.

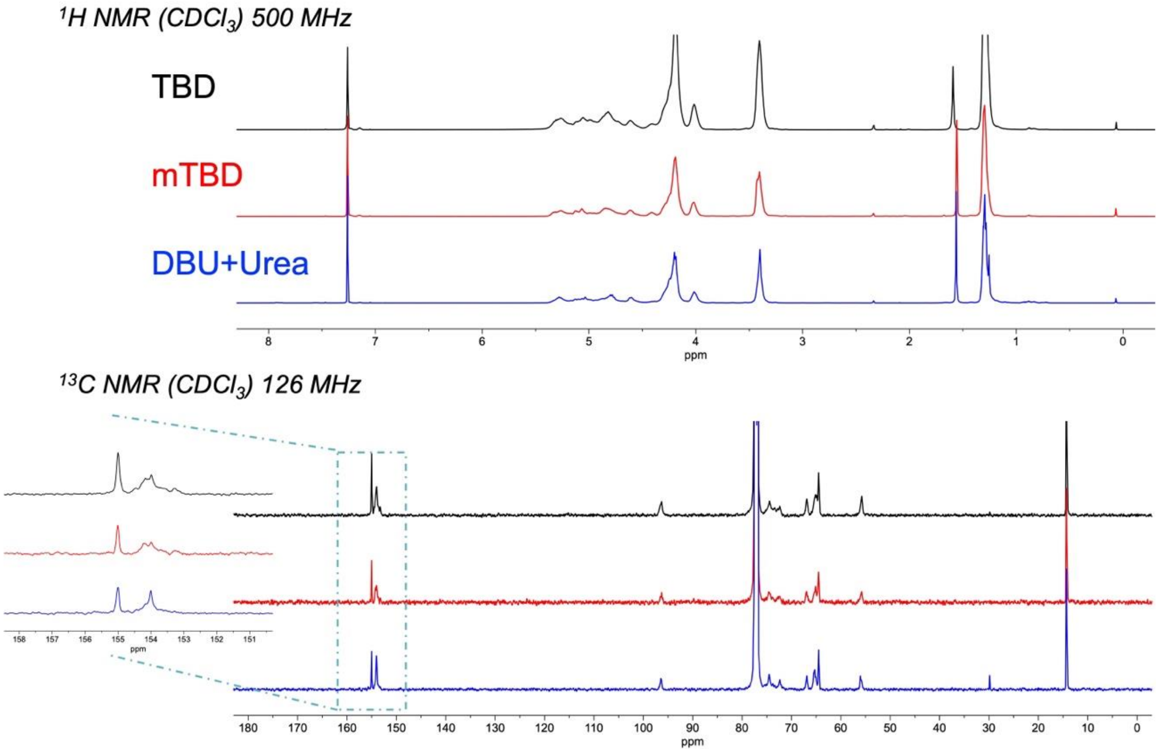

Figure S32. Comparison of ^{1}H NMR (500 MHz) and ^{13}C NMR (126 MHz) spectra of PM(EC^2)GC (**15**) *via* ROP using different catalyst in $CDCl_3$.

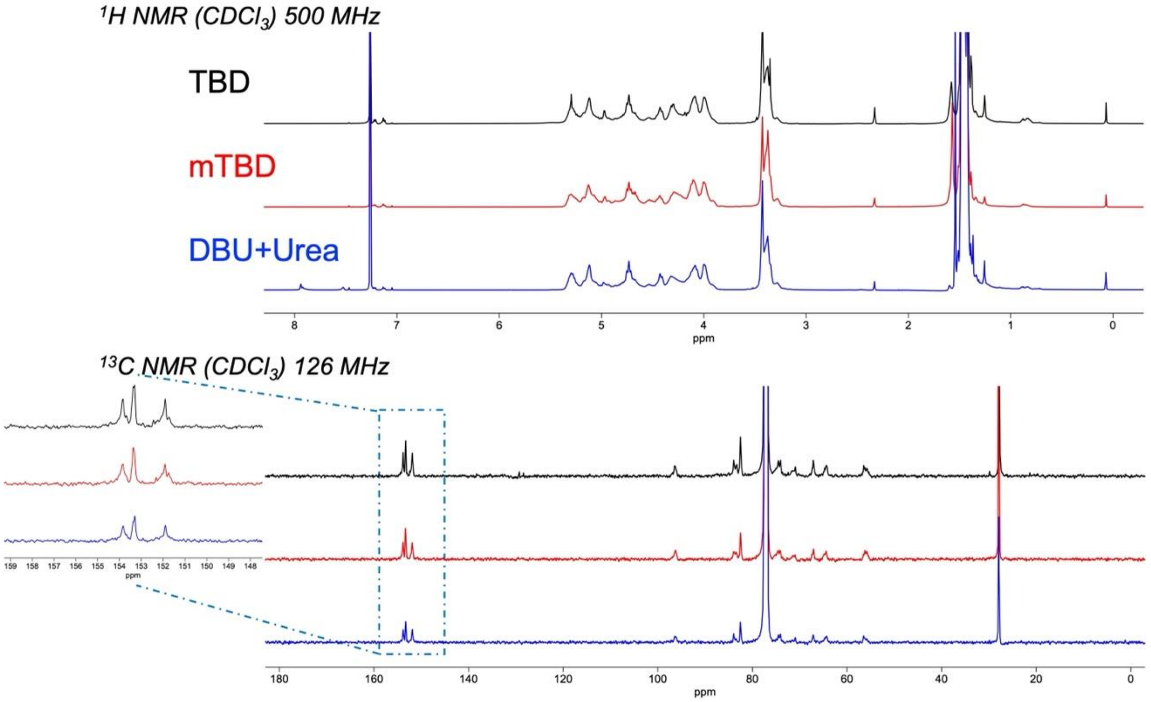

Figure S33. Comparison of ^{1}H NMR (500 MHz) and ^{13}C NMR (126 MHz) spectra of PM(*t*BuC^2)GC (**16**) *via* ROP using different catalyst in $CDCl_3$.

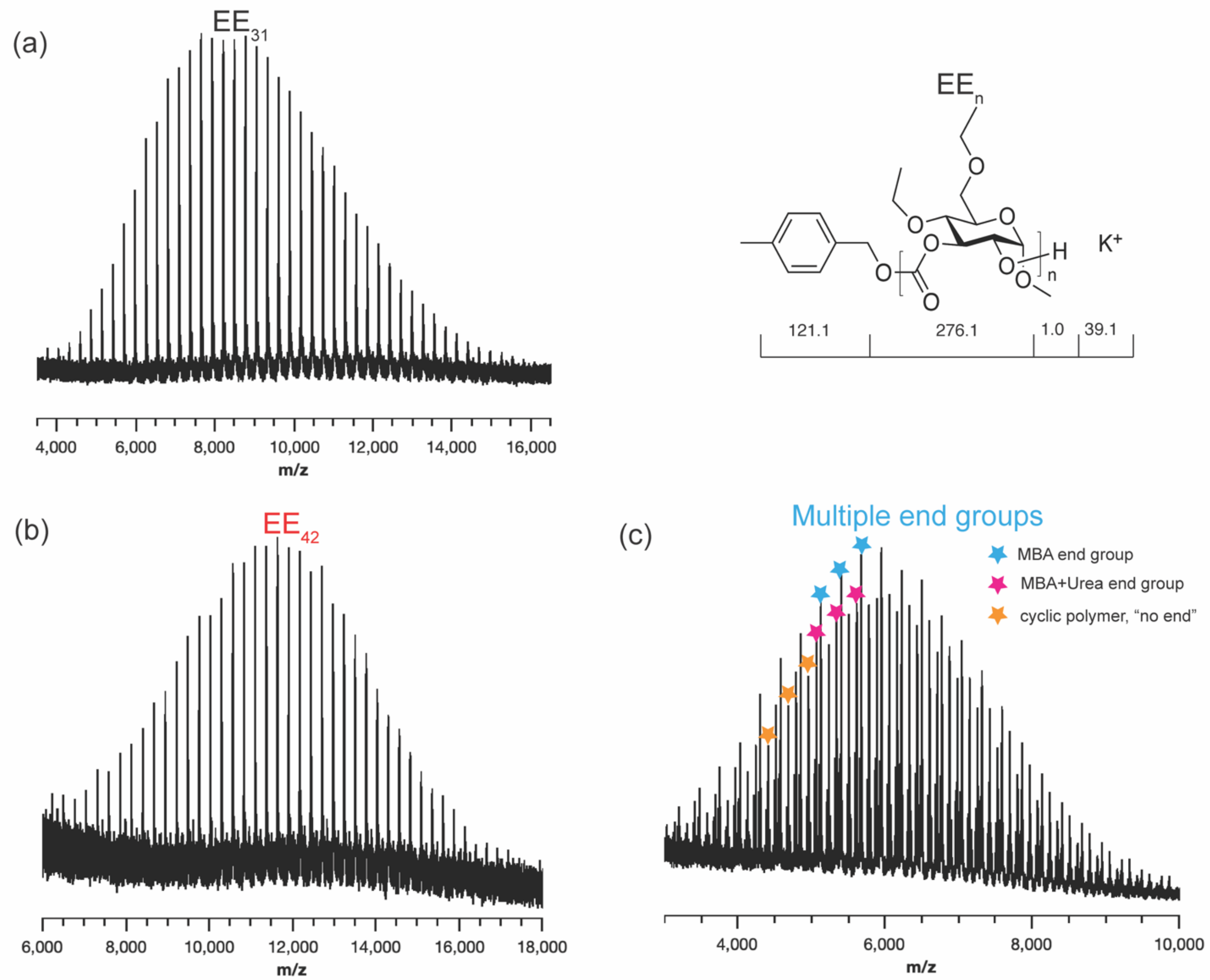

Figure S34. MALDI-TOF MS spectra of PM(EE^2)GC *via* organocatalytic ROP using (a) TBD, (b) mTBD, or (c) DBU+Urea.

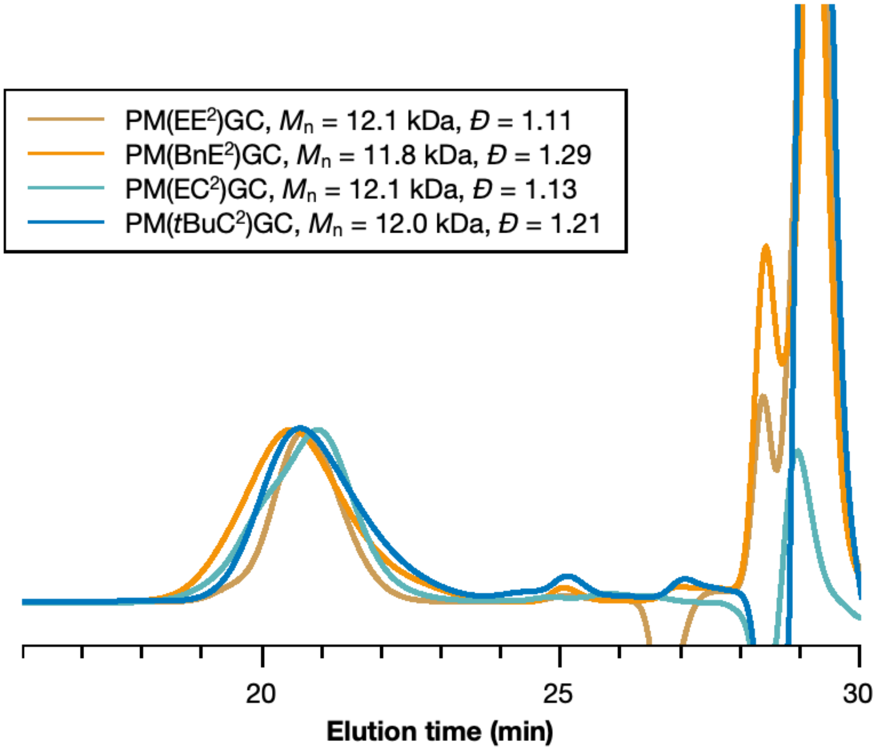

Figure S35. SEC traces of polymers for thermal analyses.

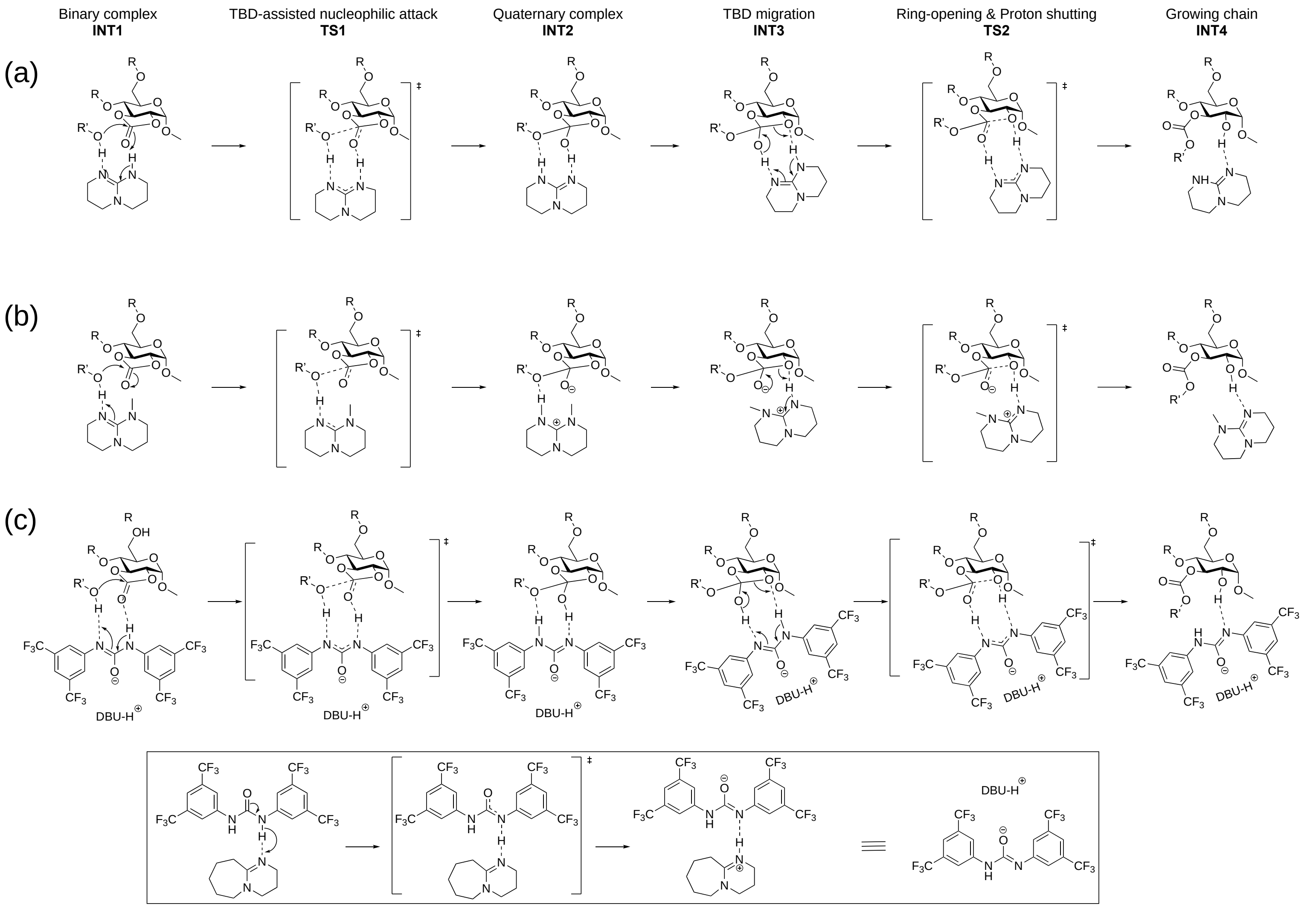

Figure S36. The initiation step of the ROP of 2,3-a-d-glucose carbonate, catalyzed by (a) TBD, (b) mTBD, and (c) DBU+Urea and initiated by MBA. R = side chain protecting group. (*There are supposed to be four possible pathways of the initiation.[1, 2] Only the initiator and catalyst attacked *syn* to the anomeric methoxy group that involved C-O2 cleavage was presented.)

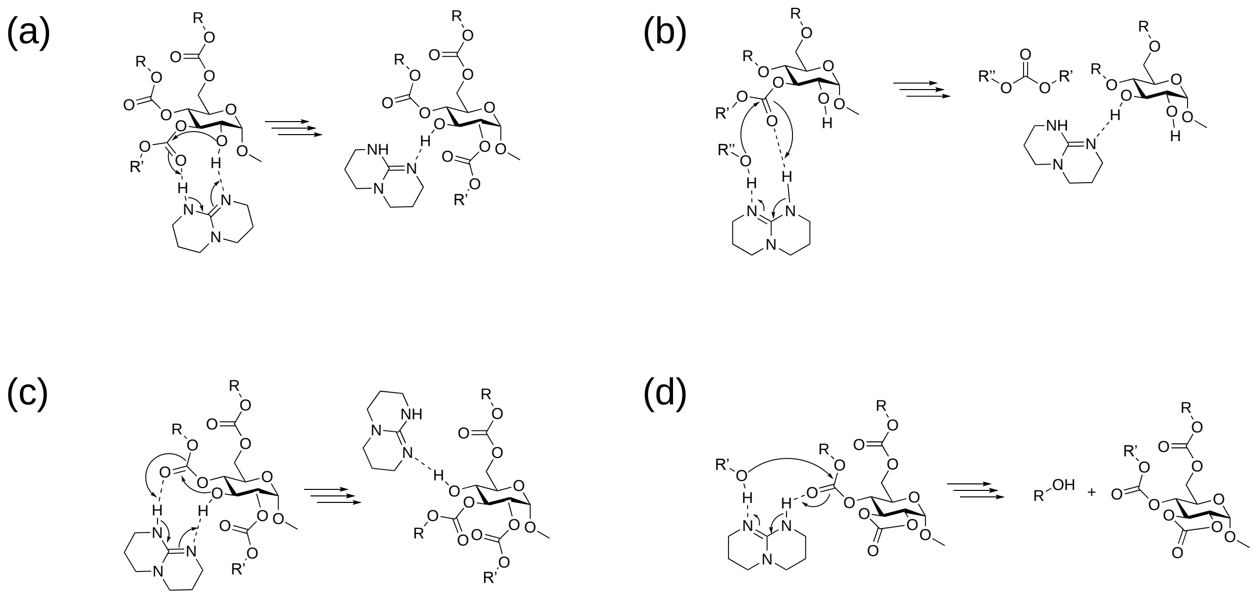

Figure S37. (a) 2- and 3- position intramolecular transcarbonylation, (b) 2- and 3- position intermolecular transcarbonylation, (c) side chain involved intramolecular transcarbonylation, (d) side chain involved intermolecular transcarbonylation.

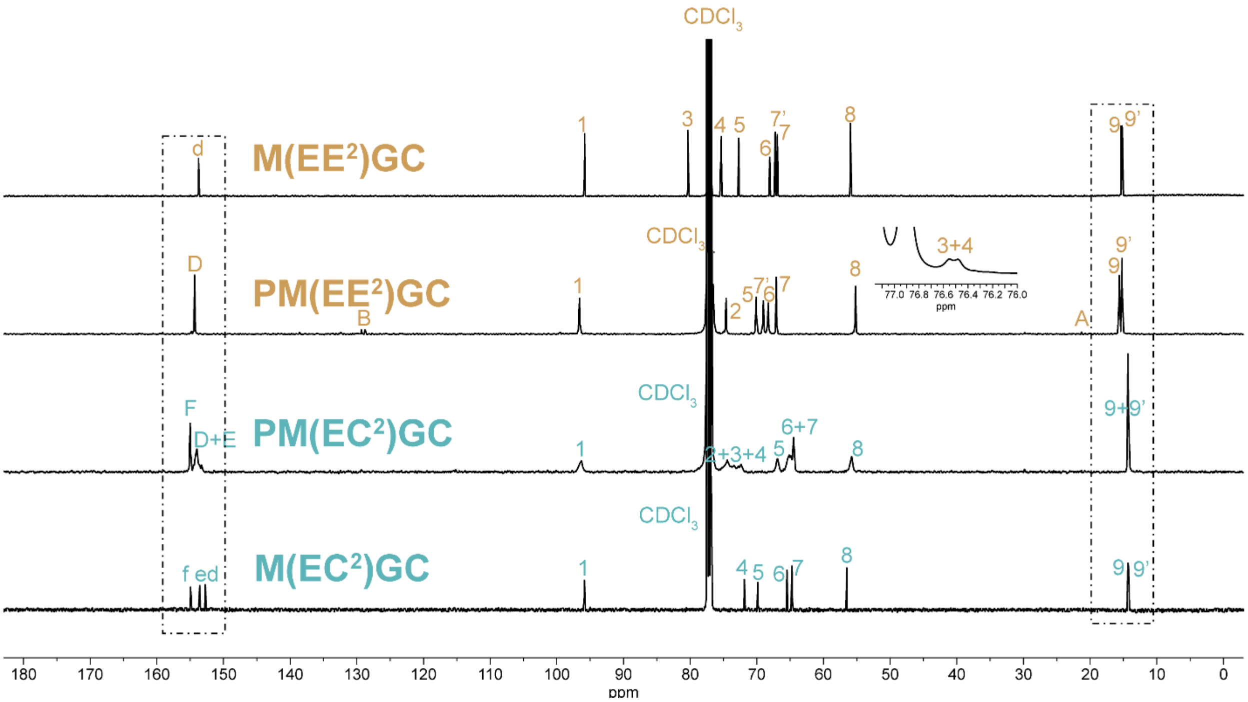

Figure S38. ^{13}C NMR (126 MHz) spectra of 1, 13, 15, and 3 (from top to bottom) in $CDCl_3$. The resonances in dashed box were enlarged in Figure 3(b).

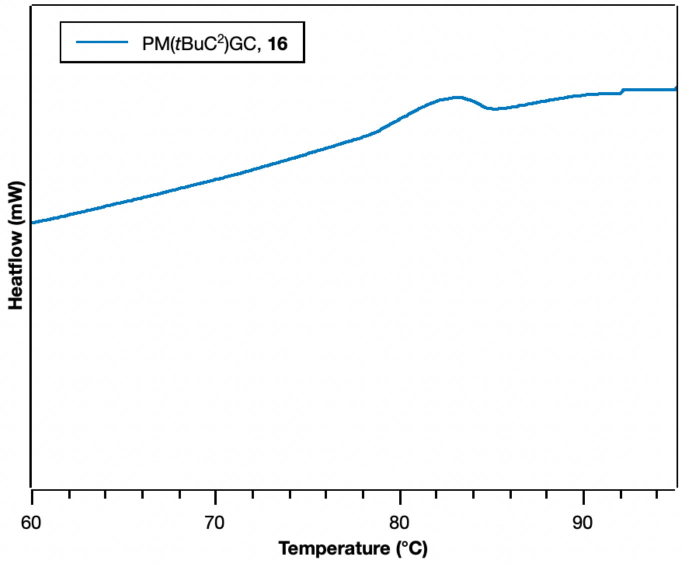

Figure S39. An enlarged DSC of Polymer **16.**

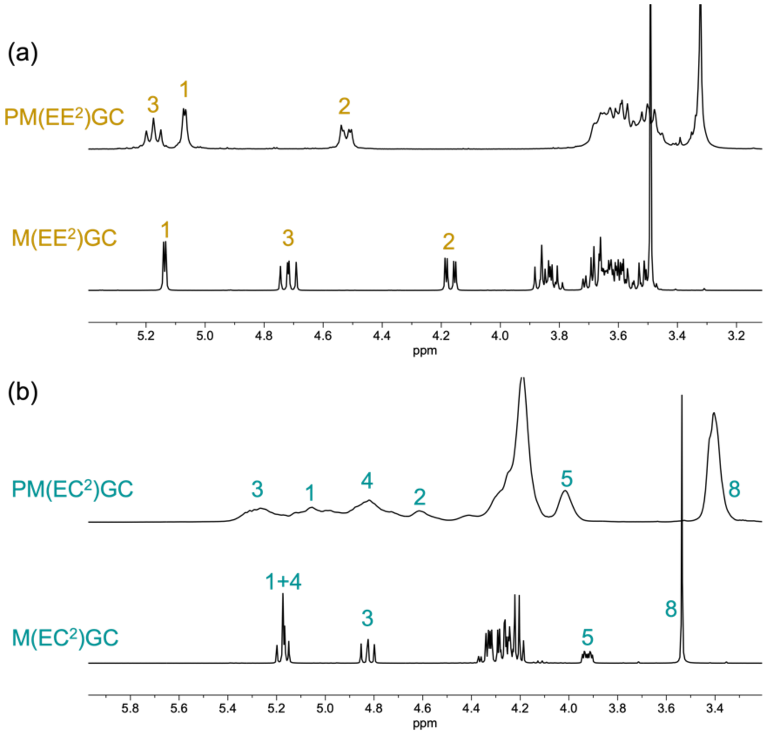

Figure S40. (a) ^{1}H NMR of PM(EE^{2})GC and M(EE^{2})GC, (b) ^{1}H NMR of PM(EC^{2})GC and M(EC^{2})GC.

References

(1) Song, Y.; Yang, X.; Shen, Y.; Dong, M.; Lin, Y.-N.; Hall, M. B.; Wooley, K. L. Invoking Side-Chain Functionality for the Mediation of Regioselectivity during Ring-Opening Polymerization of Glucose Carbonates, *J. Am. Chem. Soc.* **2020**, *142* (40), 16974-16981.

(2) Shen, Y.; Yang, X.; Song, Y.; Tran, D. K.; Wang, H.; Wilson, J.; Dong, M.; Vazquez, M.; Sun, G.; Wooley, K. L. Complexities of Regioselective Ring-Opening vs Transcarbonylation-Driven Structural Metamorphosis during Organocatalytic Polymerizations of Five-Membered Cyclic Carbonate Glucose Monomers, *JACS Au* **2022**, *2* (2), 515-521.
